# Supplementary material for: Do Different Sutures with Triclosan Have Different Antimicrobial Activities? A Pharmacodynamic Approach
Source: Antibiotics (Basel). 2022 Sep 3;11(9):1195. doi: 10.3390/antibiotics11091195 (PMC9494962; doi:10.3390/antibiotics11091195)
Supplement: Supplementary file 1 [file antibiotics-11-01195-s001.zip › antibiotics-1859012-supplementary.pdf]

Do different sutures with triclosan have different antimicrobial activities? A pharmacodynamic approach.

Frederic C. Daoud, Fatima M'Zali, Arnaud Zabala, Nicholas Moore, and Anne-Marie Rogues

Supplementary material

Figure S1 – MRSA ATCC 33592 with V+

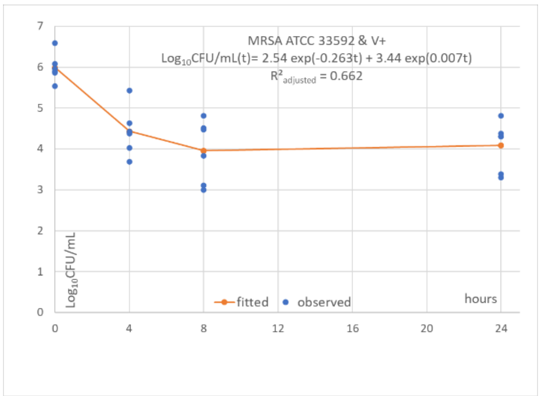

Figure S2 – MRSA ATCC 33592 with P+

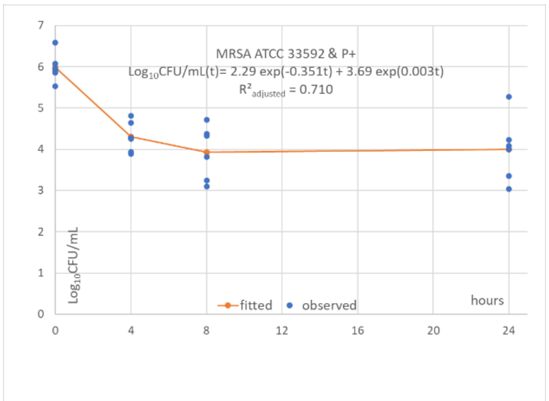

Figure S3– MRSA ATCC 33592 with M+

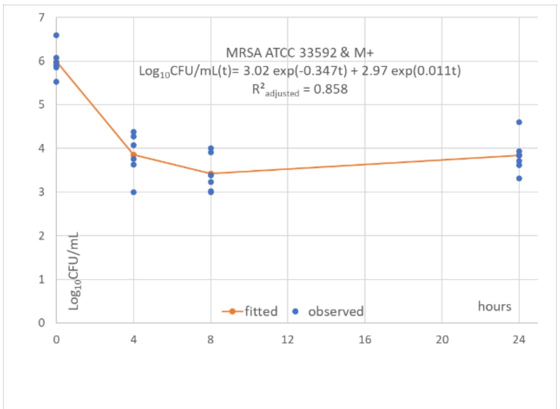

Figure S4 - MRSA clinical with V+

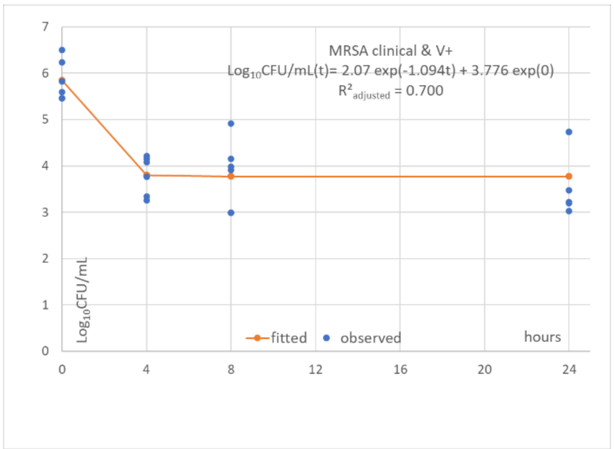

Figure S5 - MRSA clinical with P+

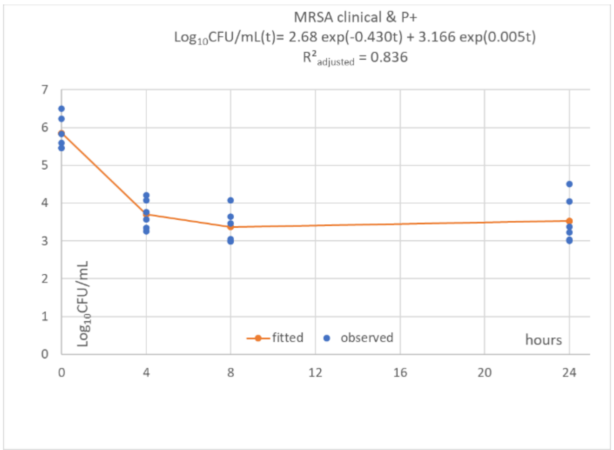

Figure S6 - MRSA clinical with M+

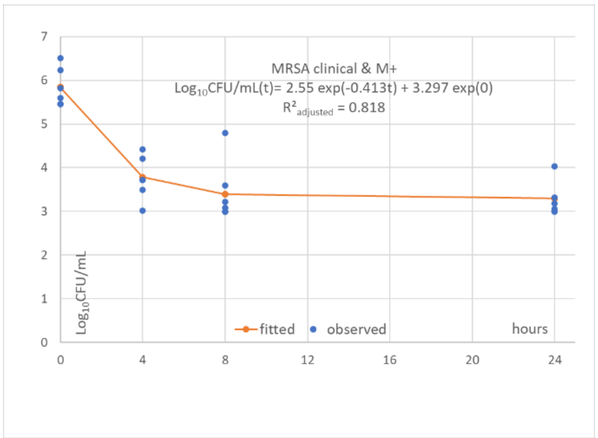

Figure S7 – C. albicans ATCC 10231 with V+

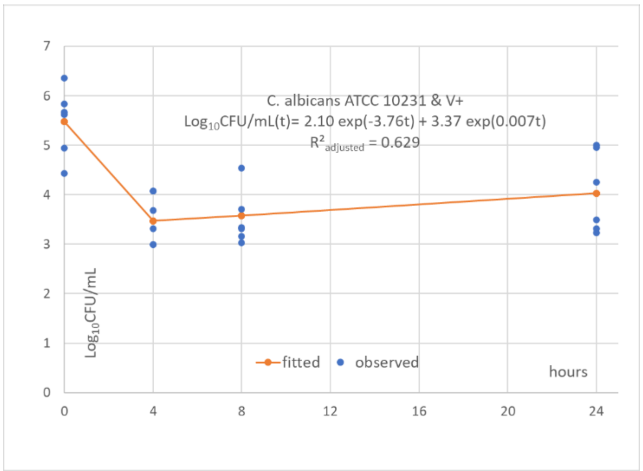

Figure S8 – C. albicans ATCC 10231 with P+

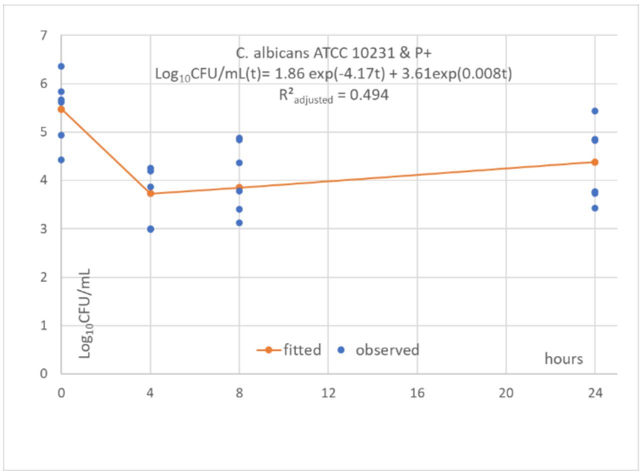

Figure S9– C. albicans ATCC 10231 with M+

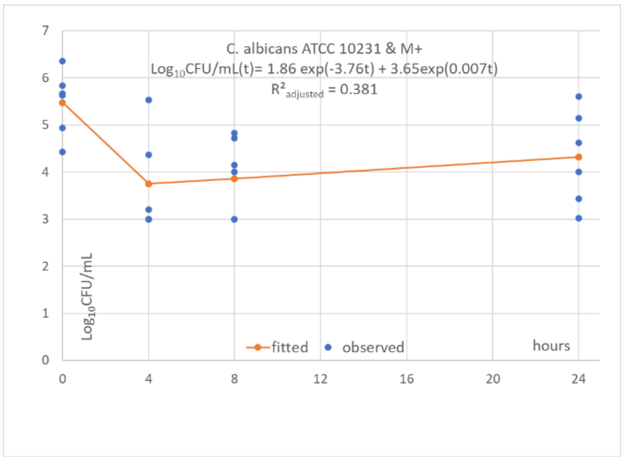

Figure S10 – C. albicans clinical with V+

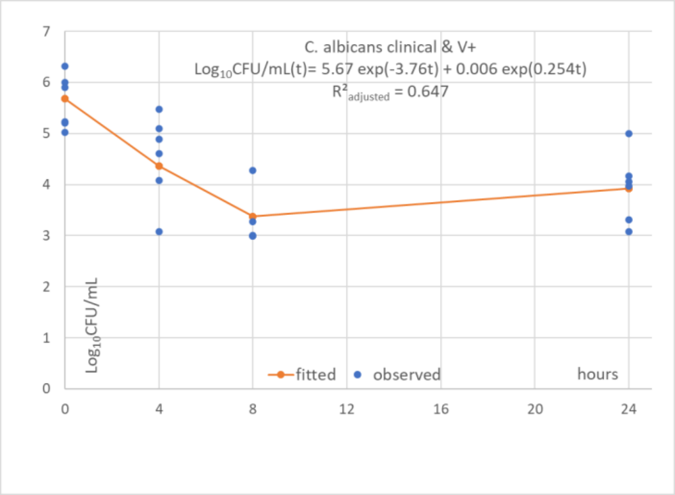

Figure S11– C. albicans clinical with P+

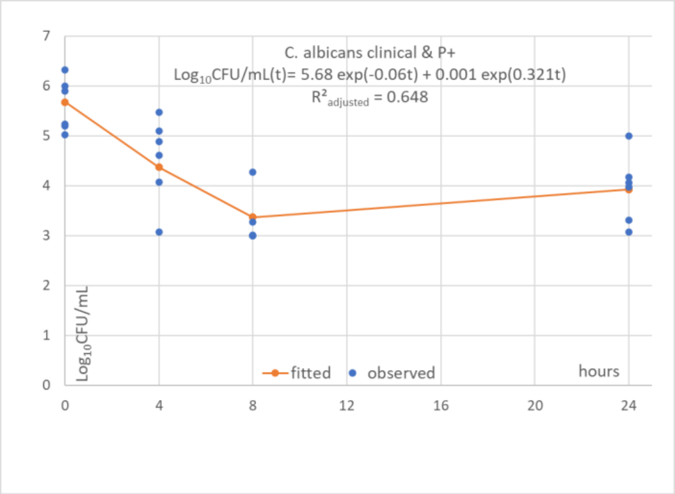

Figure S12– C. albicans clinical with M+

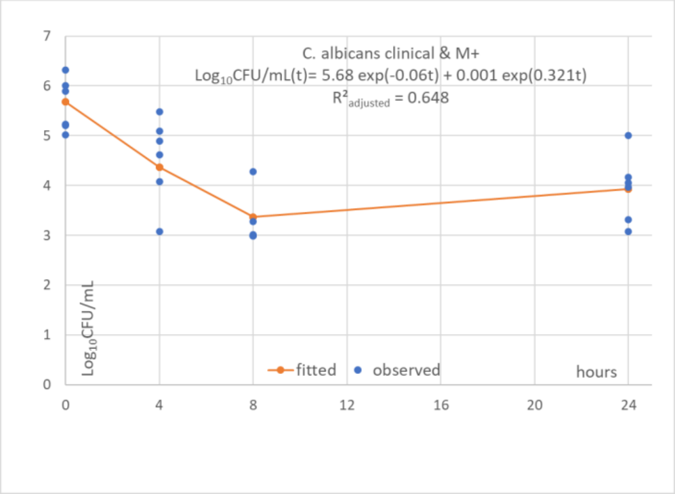

Table S1 – Pharmacodynamics raw data

id	assay	hour	order	cfu	material	triclosan	microorganism
1	n1	0	1	170000	polyglecaprone25	no	E. coli ATCC 25922
2	n1	4	2	34000000	polyglecaprone25	no	E. coli ATCC 25922
3	n1	8	3	51000000	polyglecaprone25	no	E. coli ATCC 25922
4	n1	24	4	100000000	polyglecaprone25	no	E. coli ATCC 25922
5	n2	0	1	160000	polyglecaprone25	no	E. coli ATCC 25922
6	n2	4	2	36000000	polyglecaprone25	no	E. coli ATCC 25922
7	n2	8	3	73000000	polyglecaprone25	no	E. coli ATCC 25922
8	n2	24	4	100000000	polyglecaprone25	no	E. coli ATCC 25922
9	n3	0	1	1000000	polyglecaprone25	no	E. coli ATCC 25922
10	n3	4	2	2970000	polyglecaprone25	no	E. coli ATCC 25922
11	n3	8	3	3700000	polyglecaprone25	no	E. coli ATCC 25922
12	n3	24	4	100000000	polyglecaprone25	no	E. coli ATCC 25922
13	n4	0	1	104000	polyglecaprone25	no	E. coli ATCC 25922
14	n4	4	2	214000	polyglecaprone25	no	E. coli ATCC 25922
15	n4	8	3	4860000	polyglecaprone25	no	E. coli ATCC 25922
16	n4	24	4	100000000	polyglecaprone25	no	E. coli ATCC 25922
17	n5	0	1	2100000	polyglecaprone25	no	E. coli ATCC 25922
18	n5	4	2	3700000	polyglecaprone25	no	E. coli ATCC 25922
19	n5	8	3	8200000	polyglecaprone25	no	E. coli ATCC 25922
20	n5	24	4	100000000	polyglecaprone25	no	E. coli ATCC 25922
21	n6	0	1	790000	polyglecaprone25	no	E. coli ATCC 25922
22	n6	4	2	1840000	polyglecaprone25	no	E. coli ATCC 25922
23	n6	8	3	97000000	polyglecaprone25	no	E. coli ATCC 25922
24	n6	24	4	100000000	polyglecaprone25	no	E. coli ATCC 25922
25	n1	0	1	170000	polyglactin910	no	E. coli ATCC 25922
26	n1	4	2	12000000	polyglactin910	no	E. coli ATCC 25922
27	n1	8	3	30000000	polyglactin910	no	E. coli ATCC 25922
28	n1	24	4	100000000	polyglactin910	no	E. coli ATCC 25922
29	n2	0	1	160000	polyglactin910	no	E. coli ATCC 25922
30	n2	4	2	20000000	polyglactin910	no	E. coli ATCC 25922
31	n2	8	3	60000000	polyglactin910	no	E. coli ATCC 25922
32	n2	24	4	100000000	polyglactin910	no	E. coli ATCC 25922
33	n3	0	1	1000000	polyglactin910	no	E. coli ATCC 25922
34	n3	4	2	2780000	polyglactin910	no	E. coli ATCC 25922
35	n3	8	3	30000000	polyglactin910	no	E. coli ATCC 25922
36	n3	24	4	100000000	polyglactin910	no	E. coli ATCC 25922
37	n4	0	1	104000	polyglactin910	no	E. coli ATCC 25922
38	n4	4	2	216000	polyglactin910	no	E. coli ATCC 25922
39	n4	8	3	3500000	polyglactin910	no	E. coli ATCC 25922
40	n4	24	4	100000000	polyglactin910	no	E. coli ATCC 25922
41	n5	0	1	2100000	polyglactin910	no	E. coli ATCC 25922
42	n5	4	2	3540000	polyglactin910	no	E. coli ATCC 25922
43	n5	8	3	4160000	polyglactin910	no	E. coli ATCC 25922
44	n5	24	4	100000000	polyglactin910	no	E. coli ATCC 25922
45	n6	0	1	790000	polyglactin910	no	E. coli ATCC 25922
46	n6	4	2	2040000	polyglactin910	no	E. coli ATCC 25922
47	n6	8	3	72000000	polyglactin910	no	E. coli ATCC 25922
48	n6	24	4	100000000	polyglactin910	no	E. coli ATCC 25922
49	n1	0	1	170000	polydioxanone	no	E. coli ATCC 25922
50	n1	4	2	45000000	polydioxanone	no	E. coli ATCC 25922
51	n1	8	3	71000000	polydioxanone	no	E. coli ATCC 25922
52	n1	24	4	100000000	polydioxanone	no	E. coli ATCC 25922
53	n2	0	1	160000	polydioxanone	no	E. coli ATCC 25922
54	n2	4	2	55000000	polydioxanone	no	E. coli ATCC 25922
55	n2	8	3	80000000	polydioxanone	no	E. coli ATCC 25922
56	n2	24	4	100000000	polydioxanone	no	E. coli ATCC 25922
57	n3	0	1	1000000	polydioxanone	no	E. coli ATCC 25922
58	n3	4	2	3650000	polydioxanone	no	E. coli ATCC 25922
59	n3	8	3	10400000	polydioxanone	no	E. coli ATCC 25922

60	n3	24	4	100000000	polydioxanone	no	E. coli ATCC 25922
61	n4	0	1	104000	polydioxanone	no	E. coli ATCC 25922
62	n4	4	2	189000	polydioxanone	no	E. coli ATCC 25922
63	n4	8	3	5660000	polydioxanone	no	E. coli ATCC 25922
64	n4	24	4	100000000	polydioxanone	no	E. coli ATCC 25922
65	n5	0	1	2100000	polydioxanone	no	E. coli ATCC 25922
66	n5	4	2	4580000	polydioxanone	no	E. coli ATCC 25922
67	n5	8	3	7170000	polydioxanone	no	E. coli ATCC 25922
68	n5	24	4	100000000	polydioxanone	no	E. coli ATCC 25922
69	n6	0	1	790000	polydioxanone	no	E. coli ATCC 25922
70	n6	4	2	1240000	polydioxanone	no	E. coli ATCC 25922
71	n6	8	3	5460000	polydioxanone	no	E. coli ATCC 25922
72	n6	24	4	100000000	polydioxanone	no	E. coli ATCC 25922
73	n1	0	1	170000	polyglecaprone25	yes	E. coli ATCC 25922
74	n1	4	2	3400	polyglecaprone25	yes	E. coli ATCC 25922
75	n1	8	3	6300	polyglecaprone25	yes	E. coli ATCC 25922
76	n1	24	4	91000	polyglecaprone25	yes	E. coli ATCC 25922
77	n2	0	1	160000	polyglecaprone25	yes	E. coli ATCC 25922
78	n2	4	2	990	polyglecaprone25	yes	E. coli ATCC 25922
79	n2	8	3	990	polyglecaprone25	yes	E. coli ATCC 25922
80	n2	24	4	990	polyglecaprone25	yes	E. coli ATCC 25922
81	n3	0	1	1000000	polyglecaprone25	yes	E. coli ATCC 25922
82	n3	4	2	280000	polyglecaprone25	yes	E. coli ATCC 25922
83	n3	8	3	990	polyglecaprone25	yes	E. coli ATCC 25922
84	n3	24	4	50000	polyglecaprone25	yes	E. coli ATCC 25922
85	n4	0	1	104000	polyglecaprone25	yes	E. coli ATCC 25922
86	n4	4	2	18000	polyglecaprone25	yes	E. coli ATCC 25922
87	n4	8	3	4120	polyglecaprone25	yes	E. coli ATCC 25922
88	n4	24	4	60000	polyglecaprone25	yes	E. coli ATCC 25922
89	n5	0	1	2100000	polyglecaprone25	yes	E. coli ATCC 25922
90	n5	4	2	20000	polyglecaprone25	yes	E. coli ATCC 25922
91	n5	8	3	990	polyglecaprone25	yes	E. coli ATCC 25922
92	n5	24	4	990	polyglecaprone25	yes	E. coli ATCC 25922
93	n6	0	1	790000	polyglecaprone25	yes	E. coli ATCC 25922
94	n6	4	2	15800	polyglecaprone25	yes	E. coli ATCC 25922
95	n6	8	3	990	polyglecaprone25	yes	E. coli ATCC 25922
96	n6	24	4	990	polyglecaprone25	yes	E. coli ATCC 25922
97	n1	0	1	170000	polyglactin910	yes	E. coli ATCC 25922
98	n1	4	2	1200	polyglactin910	yes	E. coli ATCC 25922
99	n1	8	3	1900	polyglactin910	yes	E. coli ATCC 25922
100	n1	24	4	100000	polyglactin910	yes	E. coli ATCC 25922
101	n2	0	1	160000	polyglactin910	yes	E. coli ATCC 25922
102	n2	4	2	12000	polyglactin910	yes	E. coli ATCC 25922
103	n2	8	3	19000	polyglactin910	yes	E. coli ATCC 25922
104	n2	24	4	11400	polyglactin910	yes	E. coli ATCC 25922
105	n3	0	1	1000000	polyglactin910	yes	E. coli ATCC 25922
106	n3	4	2	300000	polyglactin910	yes	E. coli ATCC 25922
107	n3	8	3	990	polyglactin910	yes	E. coli ATCC 25922
108	n3	24	4	14700	polyglactin910	yes	E. coli ATCC 25922
109	n4	0	1	104000	polyglactin910	yes	E. coli ATCC 25922
110	n4	4	2	77000	polyglactin910	yes	E. coli ATCC 25922
111	n4	8	3	990	polyglactin910	yes	E. coli ATCC 25922
112	n4	24	4	9300	polyglactin910	yes	E. coli ATCC 25922
113	n5	0	1	2100000	polyglactin910	yes	E. coli ATCC 25922
114	n5	4	2	41000	polyglactin910	yes	E. coli ATCC 25922
115	n5	8	3	1020	polyglactin910	yes	E. coli ATCC 25922
116	n5	24	4	2070	polyglactin910	yes	E. coli ATCC 25922
117	n6	0	1	790000	polyglactin910	yes	E. coli ATCC 25922
118	n6	4	2	124000	polyglactin910	yes	E. coli ATCC 25922
119	n6	8	3	990	polyglactin910	yes	E. coli ATCC 25922
120	n6	24	4	1190	polyglactin910	yes	E. coli ATCC 25922
121	n1	0	1	170000	polydioxanone	yes	E. coli ATCC 25922

122	n1	4	2	4500	polydioxanone	yes	E. coli ATCC 25922
123	n1	8	3	3700	polydioxanone	yes	E. coli ATCC 25922
124	n1	24	4	32000	polydioxanone	yes	E. coli ATCC 25922
125	n2	0	1	160000	polydioxanone	yes	E. coli ATCC 25922
126	n2	4	2	990	polydioxanone	yes	E. coli ATCC 25922
127	n2	8	3	1000	polydioxanone	yes	E. coli ATCC 25922
128	n2	24	4	5500	polydioxanone	yes	E. coli ATCC 25922
129	n3	0	1	1000000	polydioxanone	yes	E. coli ATCC 25922
130	n3	4	2	150000	polydioxanone	yes	E. coli ATCC 25922
131	n3	8	3	1780	polydioxanone	yes	E. coli ATCC 25922
132	n3	24	4	11000	polydioxanone	yes	E. coli ATCC 25922
133	n4	0	1	104000	polydioxanone	yes	E. coli ATCC 25922
134	n4	4	2	7100	polydioxanone	yes	E. coli ATCC 25922
135	n4	8	3	990	polydioxanone	yes	E. coli ATCC 25922
136	n4	24	4	2200	polydioxanone	yes	E. coli ATCC 25922
137	n5	0	1	2100000	polydioxanone	yes	E. coli ATCC 25922
138	n5	4	2	2000	polydioxanone	yes	E. coli ATCC 25922
139	n5	8	3	990	polydioxanone	yes	E. coli ATCC 25922
140	n5	24	4	1680	polydioxanone	yes	E. coli ATCC 25922
141	n6	0	1	790000	polydioxanone	yes	E. coli ATCC 25922
142	n6	4	2	11400	polydioxanone	yes	E. coli ATCC 25922
143	n6	8	3	990	polydioxanone	yes	E. coli ATCC 25922
144	n6	24	4	2400	polydioxanone	yes	E. coli ATCC 25922
145	n1	0	1	780000	polyglecaprone25	no	E. coli ESBL BAA-2326
146	n1	4	2	14000000	polyglecaprone25	no	E. coli ESBL BAA-2326
147	n1	8	3	49000000	polyglecaprone25	no	E. coli ESBL BAA-2326
148	n1	24	4	91000000	polyglecaprone25	no	E. coli ESBL BAA-2326
149	n2	0	1	2800000	polyglecaprone25	no	E. coli ESBL BAA-2326
150	n2	4	2	4700000	polyglecaprone25	no	E. coli ESBL BAA-2326
151	n2	8	3	7000000	polyglecaprone25	no	E. coli ESBL BAA-2326
152	n2	24	4	100000000	polyglecaprone25	no	E. coli ESBL BAA-2326
153	n3	0	1	1200000	polyglecaprone25	no	E. coli ESBL BAA-2326
154	n3	4	2	2700000	polyglecaprone25	no	E. coli ESBL BAA-2326
155	n3	8	3	4800000	polyglecaprone25	no	E. coli ESBL BAA-2326
156	n3	24	4	100000000	polyglecaprone25	no	E. coli ESBL BAA-2326
157	n4	0	1	464000	polyglecaprone25	no	E. coli ESBL BAA-2326
158	n4	4	2	821000	polyglecaprone25	no	E. coli ESBL BAA-2326
159	n4	8	3	3200000	polyglecaprone25	no	E. coli ESBL BAA-2326
160	n4	24	4	100000000	polyglecaprone25	no	E. coli ESBL BAA-2326
161	n5	0	1	1100000	polyglecaprone25	no	E. coli ESBL BAA-2326
162	n5	4	2	2300000	polyglecaprone25	no	E. coli ESBL BAA-2326
163	n5	8	3	6700000	polyglecaprone25	no	E. coli ESBL BAA-2326
164	n5	24	4	100000000	polyglecaprone25	no	E. coli ESBL BAA-2326
165	n6	0	1	980000	polyglecaprone25	no	E. coli ESBL BAA-2326
166	n6	4	2	1900000	polyglecaprone25	no	E. coli ESBL BAA-2326
167	n6	8	3	4240000	polyglecaprone25	no	E. coli ESBL BAA-2326
168	n6	24	4	100000000	polyglecaprone25	no	E. coli ESBL BAA-2326
169	n1	0	1	780000	polyglactin910	no	E. coli ESBL BAA-2326
170	n1	4	2	1000000	polyglactin910	no	E. coli ESBL BAA-2326
171	n1	8	3	50000000	polyglactin910	no	E. coli ESBL BAA-2326
172	n1	24	4	100000000	polyglactin910	no	E. coli ESBL BAA-2326
173	n2	0	1	2800000	polyglactin910	no	E. coli ESBL BAA-2326
174	n2	4	2	5500000	polyglactin910	no	E. coli ESBL BAA-2326
175	n2	8	3	8900000	polyglactin910	no	E. coli ESBL BAA-2326
176	n2	24	4	100000000	polyglactin910	no	E. coli ESBL BAA-2326
177	n3	0	1	1200000	polyglactin910	no	E. coli ESBL BAA-2326
178	n3	4	2	3980000	polyglactin910	no	E. coli ESBL BAA-2326
179	n3	8	3	7790000	polyglactin910	no	E. coli ESBL BAA-2326
180	n3	24	4	100000000	polyglactin910	no	E. coli ESBL BAA-2326
181	n4	0	1	464000	polyglactin910	no	E. coli ESBL BAA-2326
182	n4	4	2	8560000	polyglactin910	no	E. coli ESBL BAA-2326
183	n4	8	3	12600000	polyglactin910	no	E. coli ESBL BAA-2326

184	n4	24	4	100000000	polyglactin910	no	E. coli ESBL BAA-2326
185	n5	0	1	1100000	polyglactin910	no	E. coli ESBL BAA-2326
186	n5	4	2	2350000	polyglactin910	no	E. coli ESBL BAA-2326
187	n5	8	3	4840000	polyglactin910	no	E. coli ESBL BAA-2326
188	n5	24	4	100000000	polyglactin910	no	E. coli ESBL BAA-2326
189	n6	0	1	980000	polyglactin910	no	E. coli ESBL BAA-2326
190	n6	4	2	1180000	polyglactin910	no	E. coli ESBL BAA-2326
191	n6	8	3	4120000	polyglactin910	no	E. coli ESBL BAA-2326
192	n6	24	4	100000000	polyglactin910	no	E. coli ESBL BAA-2326
193	n1	0	1	780000	polydioxanone	no	E. coli ESBL BAA-2326
194	n1	4	2	980000	polydioxanone	no	E. coli ESBL BAA-2326
195	n1	8	3	13000000	polydioxanone	no	E. coli ESBL BAA-2326
196	n1	24	4	32000000	polydioxanone	no	E. coli ESBL BAA-2326
197	n2	0	1	2800000	polydioxanone	no	E. coli ESBL BAA-2326
198	n2	4	2	3300000	polydioxanone	no	E. coli ESBL BAA-2326
199	n2	8	3	5000000	polydioxanone	no	E. coli ESBL BAA-2326
200	n2	24	4	100000000	polydioxanone	no	E. coli ESBL BAA-2326
201	n3	0	1	1200000	polydioxanone	no	E. coli ESBL BAA-2326
202	n3	4	2	2020000	polydioxanone	no	E. coli ESBL BAA-2326
203	n3	8	3	8600000	polydioxanone	no	E. coli ESBL BAA-2326
204	n3	24	4	100000000	polydioxanone	no	E. coli ESBL BAA-2326
205	n4	0	1	464000	polydioxanone	no	E. coli ESBL BAA-2326
206	n4	4	2	7230000	polydioxanone	no	E. coli ESBL BAA-2326
207	n4	8	3	10400000	polydioxanone	no	E. coli ESBL BAA-2326
208	n4	24	4	100000000	polydioxanone	no	E. coli ESBL BAA-2326
209	n5	0	1	1100000	polydioxanone	no	E. coli ESBL BAA-2326
210	n5	4	2	4030000	polydioxanone	no	E. coli ESBL BAA-2326
211	n5	8	3	9180000	polydioxanone	no	E. coli ESBL BAA-2326
212	n5	24	4	100000000	polydioxanone	no	E. coli ESBL BAA-2326
213	n6	0	1	980000	polydioxanone	no	E. coli ESBL BAA-2326
214	n6	4	2	2800000	polydioxanone	no	E. coli ESBL BAA-2326
215	n6	8	3	4620000	polydioxanone	no	E. coli ESBL BAA-2326
216	n6	24	4	100000000	polydioxanone	no	E. coli ESBL BAA-2326
217	n1	0	1	780000	polyglecaprone25	yes	E. coli ESBL BAA-2326
218	n1	4	2	14000	polyglecaprone25	yes	E. coli ESBL BAA-2326
219	n1	8	3	23000	polyglecaprone25	yes	E. coli ESBL BAA-2326
220	n1	24	4	91000	polyglecaprone25	yes	E. coli ESBL BAA-2326
221	n2	0	1	2800000	polyglecaprone25	yes	E. coli ESBL BAA-2326
222	n2	4	2	22000	polyglecaprone25	yes	E. coli ESBL BAA-2326
223	n2	8	3	71000	polyglecaprone25	yes	E. coli ESBL BAA-2326
224	n2	24	4	10000	polyglecaprone25	yes	E. coli ESBL BAA-2326
225	n3	0	1	1200000	polyglecaprone25	yes	E. coli ESBL BAA-2326
226	n3	4	2	54000	polyglecaprone25	yes	E. coli ESBL BAA-2326
227	n3	8	3	2140	polyglecaprone25	yes	E. coli ESBL BAA-2326
228	n3	24	4	2000	polyglecaprone25	yes	E. coli ESBL BAA-2326
229	n4	0	1	464000	polyglecaprone25	yes	E. coli ESBL BAA-2326
230	n4	4	2	990	polyglecaprone25	yes	E. coli ESBL BAA-2326
231	n4	8	3	990	polyglecaprone25	yes	E. coli ESBL BAA-2326
232	n4	24	4	1040	polyglecaprone25	yes	E. coli ESBL BAA-2326
233	n5	0	1	1100000	polyglecaprone25	yes	E. coli ESBL BAA-2326
234	n5	4	2	107000	polyglecaprone25	yes	E. coli ESBL BAA-2326
235	n5	8	3	67400	polyglecaprone25	yes	E. coli ESBL BAA-2326
236	n5	24	4	9270	polyglecaprone25	yes	E. coli ESBL BAA-2326
237	n6	0	1	980000	polyglecaprone25	yes	E. coli ESBL BAA-2326
238	n6	4	2	41200	polyglecaprone25	yes	E. coli ESBL BAA-2326
239	n6	8	3	1040	polyglecaprone25	yes	E. coli ESBL BAA-2326
240	n6	24	4	1460	polyglecaprone25	yes	E. coli ESBL BAA-2326
241	n1	0	1	780000	polyglactin910	yes	E. coli ESBL BAA-2326
242	n1	4	2	10000	polyglactin910	yes	E. coli ESBL BAA-2326
243	n1	8	3	14000	polyglactin910	yes	E. coli ESBL BAA-2326
244	n1	24	4	10000	polyglactin910	yes	E. coli ESBL BAA-2326
245	n2	0	1	2800000	polyglactin910	yes	E. coli ESBL BAA-2326

246	n2	4	2	13000	polyglactin910	yes	E. coli ESBL BAA-2326
247	n2	8	3	84000	polyglactin910	yes	E. coli ESBL BAA-2326
248	n2	24	4	14100	polyglactin910	yes	E. coli ESBL BAA-2326
249	n3	0	1	1200000	polyglactin910	yes	E. coli ESBL BAA-2326
250	n3	4	2	236000	polyglactin910	yes	E. coli ESBL BAA-2326
251	n3	8	3	990	polyglactin910	yes	E. coli ESBL BAA-2326
252	n3	24	4	4900	polyglactin910	yes	E. coli ESBL BAA-2326
253	n4	0	1	464000	polyglactin910	yes	E. coli ESBL BAA-2326
254	n4	4	2	990	polyglactin910	yes	E. coli ESBL BAA-2326
255	n4	8	3	990	polyglactin910	yes	E. coli ESBL BAA-2326
256	n4	24	4	1950	polyglactin910	yes	E. coli ESBL BAA-2326
257	n5	0	1	1100000	polyglactin910	yes	E. coli ESBL BAA-2326
258	n5	4	2	157000	polyglactin910	yes	E. coli ESBL BAA-2326
259	n5	8	3	990	polyglactin910	yes	E. coli ESBL BAA-2326
260	n5	24	4	9120	polyglactin910	yes	E. coli ESBL BAA-2326
261	n6	0	1	980000	polyglactin910	yes	E. coli ESBL BAA-2326
262	n6	4	2	71200	polyglactin910	yes	E. coli ESBL BAA-2326
263	n6	8	3	1430	polyglactin910	yes	E. coli ESBL BAA-2326
264	n6	24	4	2600	polyglactin910	yes	E. coli ESBL BAA-2326
265	n1	0	1	780000	polydioxanone	yes	E. coli ESBL BAA-2326
266	n1	4	2	9800	polydioxanone	yes	E. coli ESBL BAA-2326
267	n1	8	3	16000	polydioxanone	yes	E. coli ESBL BAA-2326
268	n1	24	4	320000	polydioxanone	yes	E. coli ESBL BAA-2326
269	n2	0	1	2800000	polydioxanone	yes	E. coli ESBL BAA-2326
270	n2	4	2	84000	polydioxanone	yes	E. coli ESBL BAA-2326
271	n2	8	3	21000	polydioxanone	yes	E. coli ESBL BAA-2326
272	n2	24	4	83000	polydioxanone	yes	E. coli ESBL BAA-2326
273	n3	0	1	1200000	polydioxanone	yes	E. coli ESBL BAA-2326
274	n3	4	2	62000	polydioxanone	yes	E. coli ESBL BAA-2326
275	n3	8	3	12400	polydioxanone	yes	E. coli ESBL BAA-2326
276	n3	24	4	19800	polydioxanone	yes	E. coli ESBL BAA-2326
277	n4	0	1	464000	polydioxanone	yes	E. coli ESBL BAA-2326
278	n4	4	2	18400	polydioxanone	yes	E. coli ESBL BAA-2326
279	n4	8	3	2600	polydioxanone	yes	E. coli ESBL BAA-2326
280	n4	24	4	990	polydioxanone	yes	E. coli ESBL BAA-2326
281	n5	0	1	1100000	polydioxanone	yes	E. coli ESBL BAA-2326
282	n5	4	2	26500	polydioxanone	yes	E. coli ESBL BAA-2326
283	n5	8	3	990	polydioxanone	yes	E. coli ESBL BAA-2326
284	n5	24	4	1320	polydioxanone	yes	E. coli ESBL BAA-2326
285	n6	0	1	980000	polydioxanone	yes	E. coli ESBL BAA-2326
286	n6	4	2	36700	polydioxanone	yes	E. coli ESBL BAA-2326
287	n6	8	3	990	polydioxanone	yes	E. coli ESBL BAA-2326
288	n6	24	4	3400	polydioxanone	yes	E. coli ESBL BAA-2326
289	n1	0	1	900000	polyglecaprone25	no	S. aureus ATCC 29213
290	n1	4	2	86000000	polyglecaprone25	no	S. aureus ATCC 29213
291	n1	8	3	94000000	polyglecaprone25	no	S. aureus ATCC 29213
292	n1	24	4	100000000	polyglecaprone25	no	S. aureus ATCC 29213
293	n2	0	1	550000	polyglecaprone25	no	S. aureus ATCC 29213
294	n2	4	2	67000000	polyglecaprone25	no	S. aureus ATCC 29213
295	n2	8	3	83000000	polyglecaprone25	no	S. aureus ATCC 29213
296	n2	24	4	100000000	polyglecaprone25	no	S. aureus ATCC 29213
297	n3	0	1	400000	polyglecaprone25	no	S. aureus ATCC 29213
298	n3	4	2	580000	polyglecaprone25	no	S. aureus ATCC 29213
299	n3	8	3	1000000	polyglecaprone25	no	S. aureus ATCC 29213
300	n3	24	4	100000000	polyglecaprone25	no	S. aureus ATCC 29213
301	n4	0	1	520000	polyglecaprone25	no	S. aureus ATCC 29213
302	n4	4	2	1600000	polyglecaprone25	no	S. aureus ATCC 29213
303	n4	8	3	3200000	polyglecaprone25	no	S. aureus ATCC 29213
304	n4	24	4	100000000	polyglecaprone25	no	S. aureus ATCC 29213
305	n5	0	1	340000	polyglecaprone25	no	S. aureus ATCC 29213
306	n5	4	2	940000	polyglecaprone25	no	S. aureus ATCC 29213
307	n5	8	3	1400000	polyglecaprone25	no	S. aureus ATCC 29213

308	n5	24	4	100000000	polyglecaprone25	no	S. aureus ATCC 29213
309	n6	0	1	690000	polyglecaprone25	no	S. aureus ATCC 29213
310	n6	4	2	2000000	polyglecaprone25	no	S. aureus ATCC 29213
311	n6	8	3	5100000	polyglecaprone25	no	S. aureus ATCC 29213
312	n6	24	4	100000000	polyglecaprone25	no	S. aureus ATCC 29213
313	n1	0	1	900000	polyglactin910	no	S. aureus ATCC 29213
314	n1	4	2	64000000	polyglactin910	no	S. aureus ATCC 29213
315	n1	8	3	78000000	polyglactin910	no	S. aureus ATCC 29213
316	n1	24	4	100000000	polyglactin910	no	S. aureus ATCC 29213
317	n2	0	1	550000	polyglactin910	no	S. aureus ATCC 29213
318	n2	4	2	70000000	polyglactin910	no	S. aureus ATCC 29213
319	n2	8	3	90000000	polyglactin910	no	S. aureus ATCC 29213
320	n2	24	4	100000000	polyglactin910	no	S. aureus ATCC 29213
321	n3	0	1	400000	polyglactin910	no	S. aureus ATCC 29213
322	n3	4	2	620000	polyglactin910	no	S. aureus ATCC 29213
323	n3	8	3	2000000	polyglactin910	no	S. aureus ATCC 29213
324	n3	24	4	100000000	polyglactin910	no	S. aureus ATCC 29213
325	n4	0	1	520000	polyglactin910	no	S. aureus ATCC 29213
326	n4	4	2	1700000	polyglactin910	no	S. aureus ATCC 29213
327	n4	8	3	2400000	polyglactin910	no	S. aureus ATCC 29213
328	n4	24	4	100000000	polyglactin910	no	S. aureus ATCC 29213
329	n5	0	1	340000	polyglactin910	no	S. aureus ATCC 29213
330	n5	4	2	590000	polyglactin910	no	S. aureus ATCC 29213
331	n5	8	3	1800000	polyglactin910	no	S. aureus ATCC 29213
332	n5	24	4	100000000	polyglactin910	no	S. aureus ATCC 29213
333	n6	0	1	690000	polyglactin910	no	S. aureus ATCC 29213
334	n6	4	2	1160000	polyglactin910	no	S. aureus ATCC 29213
335	n6	8	3	5500000	polyglactin910	no	S. aureus ATCC 29213
336	n6	24	4	100000000	polyglactin910	no	S. aureus ATCC 29213
337	n1	0	1	900000	polydioxanone	no	S. aureus ATCC 29213
338	n1	4	2	58000000	polydioxanone	no	S. aureus ATCC 29213
339	n1	8	3	81000000	polydioxanone	no	S. aureus ATCC 29213
340	n1	24	4	100000000	polydioxanone	no	S. aureus ATCC 29213
341	n2	0	1	550000	polydioxanone	no	S. aureus ATCC 29213
342	n2	4	2	60000000	polydioxanone	no	S. aureus ATCC 29213
343	n2	8	3	66000000	polydioxanone	no	S. aureus ATCC 29213
344	n2	24	4	100000000	polydioxanone	no	S. aureus ATCC 29213
345	n3	0	1	400000	polydioxanone	no	S. aureus ATCC 29213
346	n3	4	2	600000	polydioxanone	no	S. aureus ATCC 29213
347	n3	8	3	820000	polydioxanone	no	S. aureus ATCC 29213
348	n3	24	4	100000000	polydioxanone	no	S. aureus ATCC 29213
349	n4	0	1	520000	polydioxanone	no	S. aureus ATCC 29213
350	n4	4	2	1370000	polydioxanone	no	S. aureus ATCC 29213
351	n4	8	3	2470000	polydioxanone	no	S. aureus ATCC 29213
352	n4	24	4	100000000	polydioxanone	no	S. aureus ATCC 29213
353	n5	0	1	340000	polydioxanone	no	S. aureus ATCC 29213
354	n5	4	2	420000	polydioxanone	no	S. aureus ATCC 29213
355	n5	8	3	1340000	polydioxanone	no	S. aureus ATCC 29213
356	n5	24	4	100000000	polydioxanone	no	S. aureus ATCC 29213
357	n6	0	1	690000	polydioxanone	no	S. aureus ATCC 29213
358	n6	4	2	2040000	polydioxanone	no	S. aureus ATCC 29213
359	n6	8	3	146000000	polydioxanone	no	S. aureus ATCC 29213
360	n6	24	4	100000000	polydioxanone	no	S. aureus ATCC 29213
361	n1	0	1	900000	polyglecaprone25	yes	S. aureus ATCC 29213
362	n1	4	2	20000	polyglecaprone25	yes	S. aureus ATCC 29213
363	n1	8	3	34000	polyglecaprone25	yes	S. aureus ATCC 29213
364	n1	24	4	7000	polyglecaprone25	yes	S. aureus ATCC 29213
365	n2	0	1	550000	polyglecaprone25	yes	S. aureus ATCC 29213
366	n2	4	2	560000	polyglecaprone25	yes	S. aureus ATCC 29213
367	n2	8	3	38000	polyglecaprone25	yes	S. aureus ATCC 29213
368	n2	24	4	40000	polyglecaprone25	yes	S. aureus ATCC 29213
369	n3	0	1	400000	polyglecaprone25	yes	S. aureus ATCC 29213

370	n3	4	2	42000	polyglecaprone25	yes	S. aureus ATCC 29213
371	n3	8	3	14700	polyglecaprone25	yes	S. aureus ATCC 29213
372	n3	24	4	32000	polyglecaprone25	yes	S. aureus ATCC 29213
373	n4	0	1	520000	polyglecaprone25	yes	S. aureus ATCC 29213
374	n4	4	2	42000	polyglecaprone25	yes	S. aureus ATCC 29213
375	n4	8	3	2040	polyglecaprone25	yes	S. aureus ATCC 29213
376	n4	24	4	1200	polyglecaprone25	yes	S. aureus ATCC 29213
377	n5	0	1	340000	polyglecaprone25	yes	S. aureus ATCC 29213
378	n5	4	2	14800	polyglecaprone25	yes	S. aureus ATCC 29213
379	n5	8	3	2700	polyglecaprone25	yes	S. aureus ATCC 29213
380	n5	24	4	3000	polyglecaprone25	yes	S. aureus ATCC 29213
381	n6	0	1	690000	polyglecaprone25	yes	S. aureus ATCC 29213
382	n6	4	2	20600	polyglecaprone25	yes	S. aureus ATCC 29213
383	n6	8	3	1570	polyglecaprone25	yes	S. aureus ATCC 29213
384	n6	24	4	1600	polyglecaprone25	yes	S. aureus ATCC 29213
385	n1	0	1	900000	polyglactin910	yes	S. aureus ATCC 29213
386	n1	4	2	87000	polyglactin910	yes	S. aureus ATCC 29213
387	n1	8	3	92000	polyglactin910	yes	S. aureus ATCC 29213
388	n1	24	4	200000	polyglactin910	yes	S. aureus ATCC 29213
389	n2	0	1	550000	polyglactin910	yes	S. aureus ATCC 29213
390	n2	4	2	56000	polyglactin910	yes	S. aureus ATCC 29213
391	n2	8	3	49000	polyglactin910	yes	S. aureus ATCC 29213
392	n2	24	4	51000	polyglactin910	yes	S. aureus ATCC 29213
393	n3	0	1	400000	polyglactin910	yes	S. aureus ATCC 29213
394	n3	4	2	50000	polyglactin910	yes	S. aureus ATCC 29213
395	n3	8	3	24000	polyglactin910	yes	S. aureus ATCC 29213
396	n3	24	4	34000	polyglactin910	yes	S. aureus ATCC 29213
397	n4	0	1	520000	polyglactin910	yes	S. aureus ATCC 29213
398	n4	4	2	23600	polyglactin910	yes	S. aureus ATCC 29213
399	n4	8	3	13600	polyglactin910	yes	S. aureus ATCC 29213
400	n4	24	4	2470	polyglactin910	yes	S. aureus ATCC 29213
401	n5	0	1	340000	polyglactin910	yes	S. aureus ATCC 29213
402	n5	4	2	21700	polyglactin910	yes	S. aureus ATCC 29213
403	n5	8	3	7600	polyglactin910	yes	S. aureus ATCC 29213
404	n5	24	4	2140	polyglactin910	yes	S. aureus ATCC 29213
405	n6	0	1	690000	polyglactin910	yes	S. aureus ATCC 29213
406	n6	4	2	140000	polyglactin910	yes	S. aureus ATCC 29213
407	n6	8	3	16800	polyglactin910	yes	S. aureus ATCC 29213
408	n6	24	4	2950	polyglactin910	yes	S. aureus ATCC 29213
409	n1	0	1	900000	polydioxanone	yes	S. aureus ATCC 29213
410	n1	4	2	6000	polydioxanone	yes	S. aureus ATCC 29213
411	n1	8	3	7200	polydioxanone	yes	S. aureus ATCC 29213
412	n1	24	4	1200	polydioxanone	yes	S. aureus ATCC 29213
413	n2	0	1	550000	polydioxanone	yes	S. aureus ATCC 29213
414	n2	4	2	32000	polydioxanone	yes	S. aureus ATCC 29213
415	n2	8	3	3400	polydioxanone	yes	S. aureus ATCC 29213
416	n2	24	4	56000	polydioxanone	yes	S. aureus ATCC 29213
417	n3	0	1	400000	polydioxanone	yes	S. aureus ATCC 29213
418	n3	4	2	44000	polydioxanone	yes	S. aureus ATCC 29213
419	n3	8	3	20300	polydioxanone	yes	S. aureus ATCC 29213
420	n3	24	4	8100	polydioxanone	yes	S. aureus ATCC 29213
421	n4	0	1	520000	polydioxanone	yes	S. aureus ATCC 29213
422	n4	4	2	4100	polydioxanone	yes	S. aureus ATCC 29213
423	n4	8	3	1320	polydioxanone	yes	S. aureus ATCC 29213
424	n4	24	4	1170	polydioxanone	yes	S. aureus ATCC 29213
425	n5	0	1	340000	polydioxanone	yes	S. aureus ATCC 29213
426	n5	4	2	2000	polydioxanone	yes	S. aureus ATCC 29213
427	n5	8	3	1040	polydioxanone	yes	S. aureus ATCC 29213
428	n5	24	4	2760	polydioxanone	yes	S. aureus ATCC 29213
429	n6	0	1	690000	polydioxanone	yes	S. aureus ATCC 29213
430	n6	4	2	11400	polydioxanone	yes	S. aureus ATCC 29213
431	n6	8	3	6570	polydioxanone	yes	S. aureus ATCC 29213

432	n6	24	4	1760	polydioxanone	yes	S. aureus ATCC 29213
433	n1	0	1	3900000	polyglecaprone25	no	MRSA ATCC 33592
434	n1	4	2	12000000	polyglecaprone25	no	MRSA ATCC 33592
435	n1	8	3	34000000	polyglecaprone25	no	MRSA ATCC 33592
436	n1	24	4	70000000	polyglecaprone25	no	MRSA ATCC 33592
437	n2	0	1	800000	polyglecaprone25	no	MRSA ATCC 33592
438	n2	4	2	820000	polyglecaprone25	no	MRSA ATCC 33592
439	n2	8	3	850000	polyglecaprone25	no	MRSA ATCC 33592
440	n2	24	4	100000000	polyglecaprone25	no	MRSA ATCC 33592
441	n3	0	1	720000	polyglecaprone25	no	MRSA ATCC 33592
442	n3	4	2	890000	polyglecaprone25	no	MRSA ATCC 33592
443	n3	8	3	1300000	polyglecaprone25	no	MRSA ATCC 33592
444	n3	24	4	100000000	polyglecaprone25	no	MRSA ATCC 33592
445	n4	0	1	340000	polyglecaprone25	no	MRSA ATCC 33592
446	n4	4	2	541000	polyglecaprone25	no	MRSA ATCC 33592
447	n4	8	3	960000	polyglecaprone25	no	MRSA ATCC 33592
448	n4	24	4	100000000	polyglecaprone25	no	MRSA ATCC 33592
449	n5	0	1	910000	polyglecaprone25	no	MRSA ATCC 33592
450	n5	4	2	2100000	polyglecaprone25	no	MRSA ATCC 33592
451	n5	8	3	8140000	polyglecaprone25	no	MRSA ATCC 33592
452	n5	24	4	100000000	polyglecaprone25	no	MRSA ATCC 33592
453	n6	0	1	1200000	polyglecaprone25	no	MRSA ATCC 33592
454	n6	4	2	4500000	polyglecaprone25	no	MRSA ATCC 33592
455	n6	8	3	30000000	polyglecaprone25	no	MRSA ATCC 33592
456	n6	24	4	100000000	polyglecaprone25	no	MRSA ATCC 33592
457	n1	0	1	3900000	polyglactin910	no	MRSA ATCC 33592
458	n1	4	2	24000000	polyglactin910	no	MRSA ATCC 33592
459	n1	8	3	67000000	polyglactin910	no	MRSA ATCC 33592
460	n1	24	4	100000000	polyglactin910	no	MRSA ATCC 33592
461	n2	0	1	800000	polyglactin910	no	MRSA ATCC 33592
462	n2	4	2	850000	polyglactin910	no	MRSA ATCC 33592
463	n2	8	3	870000	polyglactin910	no	MRSA ATCC 33592
464	n2	24	4	100000000	polyglactin910	no	MRSA ATCC 33592
465	n3	0	1	720000	polyglactin910	no	MRSA ATCC 33592
466	n3	4	2	2900000	polyglactin910	no	MRSA ATCC 33592
467	n3	8	3	10000000	polyglactin910	no	MRSA ATCC 33592
468	n3	24	4	100000000	polyglactin910	no	MRSA ATCC 33592
469	n4	0	1	340000	polyglactin910	no	MRSA ATCC 33592
470	n4	4	2	4800000	polyglactin910	no	MRSA ATCC 33592
471	n4	8	3	16000000	polyglactin910	no	MRSA ATCC 33592
472	n4	24	4	100000000	polyglactin910	no	MRSA ATCC 33592
473	n5	0	1	910000	polyglactin910	no	MRSA ATCC 33592
474	n5	4	2	2100000	polyglactin910	no	MRSA ATCC 33592
475	n5	8	3	5400000	polyglactin910	no	MRSA ATCC 33592
476	n5	24	4	100000000	polyglactin910	no	MRSA ATCC 33592
477	n6	0	1	1200000	polyglactin910	no	MRSA ATCC 33592
478	n6	4	2	2900000	polyglactin910	no	MRSA ATCC 33592
479	n6	8	3	9120000	polyglactin910	no	MRSA ATCC 33592
480	n6	24	4	100000000	polyglactin910	no	MRSA ATCC 33592
481	n1	0	1	3900000	polydioxanone	no	MRSA ATCC 33592
482	n1	4	2	18000000	polydioxanone	no	MRSA ATCC 33592
483	n1	8	3	49000000	polydioxanone	no	MRSA ATCC 33592
484	n1	24	4	100000000	polydioxanone	no	MRSA ATCC 33592
485	n2	0	1	800000	polydioxanone	no	MRSA ATCC 33592
486	n2	4	2	870000	polydioxanone	no	MRSA ATCC 33592
487	n2	8	3	880000	polydioxanone	no	MRSA ATCC 33592
488	n2	24	4	100000000	polydioxanone	no	MRSA ATCC 33592
489	n3	0	1	720000	polydioxanone	no	MRSA ATCC 33592
490	n3	4	2	7800000	polydioxanone	no	MRSA ATCC 33592
491	n3	8	3	9900000	polydioxanone	no	MRSA ATCC 33592
492	n3	24	4	100000000	polydioxanone	no	MRSA ATCC 33592
493	n4	0	1	340000	polydioxanone	no	MRSA ATCC 33592

494	n4	4	2	810000	polydioxanone	no	MRSA ATCC 33592
495	n4	8	3	1650000	polydioxanone	no	MRSA ATCC 33592
496	n4	24	4	100000000	polydioxanone	no	MRSA ATCC 33592
497	n5	0	1	910000	polydioxanone	no	MRSA ATCC 33592
498	n5	4	2	1080000	polydioxanone	no	MRSA ATCC 33592
499	n5	8	3	5410000	polydioxanone	no	MRSA ATCC 33592
500	n5	24	4	100000000	polydioxanone	no	MRSA ATCC 33592
501	n6	0	1	1200000	polydioxanone	no	MRSA ATCC 33592
502	n6	4	2	3120000	polydioxanone	no	MRSA ATCC 33592
503	n6	8	3	7900000	polydioxanone	no	MRSA ATCC 33592
504	n6	24	4	100000000	polydioxanone	no	MRSA ATCC 33592
505	n1	0	1	3900000	polyglecaprone25	yes	MRSA ATCC 33592
506	n1	4	2	12000	polyglecaprone25	yes	MRSA ATCC 33592
507	n1	8	3	10000	polyglecaprone25	yes	MRSA ATCC 33592
508	n1	24	4	7000	polyglecaprone25	yes	MRSA ATCC 33592
509	n2	0	1	800000	polyglecaprone25	yes	MRSA ATCC 33592
510	n2	4	2	4300	polyglecaprone25	yes	MRSA ATCC 33592
511	n2	8	3	8100	polyglecaprone25	yes	MRSA ATCC 33592
512	n2	24	4	40000	polyglecaprone25	yes	MRSA ATCC 33592
513	n3	0	1	720000	polyglecaprone25	yes	MRSA ATCC 33592
514	n3	4	2	24000	polyglecaprone25	yes	MRSA ATCC 33592
515	n3	8	3	1060	polyglecaprone25	yes	MRSA ATCC 33592
516	n3	24	4	4200	polyglecaprone25	yes	MRSA ATCC 33592
517	n4	0	1	340000	polyglecaprone25	yes	MRSA ATCC 33592
518	n4	4	2	19000	polyglecaprone25	yes	MRSA ATCC 33592
519	n4	8	3	2400	polyglecaprone25	yes	MRSA ATCC 33592
520	n4	24	4	2070	polyglecaprone25	yes	MRSA ATCC 33592
521	n5	0	1	910000	polyglecaprone25	yes	MRSA ATCC 33592
522	n5	4	2	5700	polyglecaprone25	yes	MRSA ATCC 33592
523	n5	8	3	990	polyglecaprone25	yes	MRSA ATCC 33592
524	n5	24	4	8600	polyglecaprone25	yes	MRSA ATCC 33592
525	n6	0	1	1200000	polyglecaprone25	yes	MRSA ATCC 33592
526	n6	4	2	990	polyglecaprone25	yes	MRSA ATCC 33592
527	n6	8	3	1700	polyglecaprone25	yes	MRSA ATCC 33592
528	n6	24	4	5140	polyglecaprone25	yes	MRSA ATCC 33592
529	n1	0	1	3900000	polyglactin910	yes	MRSA ATCC 33592
530	n1	4	2	24000	polyglactin910	yes	MRSA ATCC 33592
531	n1	8	3	32000	polyglactin910	yes	MRSA ATCC 33592
532	n1	24	4	20000	polyglactin910	yes	MRSA ATCC 33592
533	n2	0	1	800000	polyglactin910	yes	MRSA ATCC 33592
534	n2	4	2	4900	polyglactin910	yes	MRSA ATCC 33592
535	n2	8	3	64000	polyglactin910	yes	MRSA ATCC 33592
536	n2	24	4	65000	polyglactin910	yes	MRSA ATCC 33592
537	n3	0	1	720000	polyglactin910	yes	MRSA ATCC 33592
538	n3	4	2	26400	polyglactin910	yes	MRSA ATCC 33592
539	n3	8	3	1280	polyglactin910	yes	MRSA ATCC 33592
540	n3	24	4	2000	polyglactin910	yes	MRSA ATCC 33592
541	n4	0	1	340000	polyglactin910	yes	MRSA ATCC 33592
542	n4	4	2	10650	polyglactin910	yes	MRSA ATCC 33592
543	n4	8	3	990	polyglactin910	yes	MRSA ATCC 33592
544	n4	24	4	2400	polyglactin910	yes	MRSA ATCC 33592
545	n5	0	1	910000	polyglactin910	yes	MRSA ATCC 33592
546	n5	4	2	43200	polyglactin910	yes	MRSA ATCC 33592
547	n5	8	3	6700	polyglactin910	yes	MRSA ATCC 33592
548	n5	24	4	20200	polyglactin910	yes	MRSA ATCC 33592
549	n6	0	1	1200000	polyglactin910	yes	MRSA ATCC 33592
550	n6	4	2	265000	polyglactin910	yes	MRSA ATCC 33592
551	n6	8	3	30000	polyglactin910	yes	MRSA ATCC 33592
552	n6	24	4	24000	polyglactin910	yes	MRSA ATCC 33592
553	n1	0	1	3900000	polydioxanone	yes	MRSA ATCC 33592
554	n1	4	2	18000	polydioxanone	yes	MRSA ATCC 33592
555	n1	8	3	21000	polydioxanone	yes	MRSA ATCC 33592

556	n1	24	4	12000	polydioxanone	yes	MRSA ATCC 33592
557	n2	0	1	800000	polydioxanone	yes	MRSA ATCC 33592
558	n2	4	2	19000	polydioxanone	yes	MRSA ATCC 33592
559	n2	8	3	52000	polydioxanone	yes	MRSA ATCC 33592
560	n2	24	4	190000	polydioxanone	yes	MRSA ATCC 33592
561	n3	0	1	720000	polydioxanone	yes	MRSA ATCC 33592
562	n3	4	2	43600	polydioxanone	yes	MRSA ATCC 33592
563	n3	8	3	1240	polydioxanone	yes	MRSA ATCC 33592
564	n3	24	4	1080	polydioxanone	yes	MRSA ATCC 33592
565	n4	0	1	340000	polydioxanone	yes	MRSA ATCC 33592
566	n4	4	2	8740	polydioxanone	yes	MRSA ATCC 33592
567	n4	8	3	1760	polydioxanone	yes	MRSA ATCC 33592
568	n4	24	4	2250	polydioxanone	yes	MRSA ATCC 33592
569	n5	0	1	910000	polydioxanone	yes	MRSA ATCC 33592
570	n5	4	2	7690	polydioxanone	yes	MRSA ATCC 33592
571	n5	8	3	6480	polydioxanone	yes	MRSA ATCC 33592
572	n5	24	4	10000	polydioxanone	yes	MRSA ATCC 33592
573	n6	0	1	1200000	polydioxanone	yes	MRSA ATCC 33592
574	n6	4	2	65600	polydioxanone	yes	MRSA ATCC 33592
575	n6	8	3	23700	polydioxanone	yes	MRSA ATCC 33592
576	n6	24	4	16700	polydioxanone	yes	MRSA ATCC 33592
577	n1	0	1	290000	polyglecaprone25	no	MRSA clinical strain
578	n1	4	2	33000000	polyglecaprone25	no	MRSA clinical strain
579	n1	8	3	79000000	polyglecaprone25	no	MRSA clinical strain
580	n1	24	4	100000000	polyglecaprone25	no	MRSA clinical strain
581	n2	0	1	290000	polyglecaprone25	no	MRSA clinical strain
582	n2	4	2	36000000	polyglecaprone25	no	MRSA clinical strain
583	n2	8	3	56000000	polyglecaprone25	no	MRSA clinical strain
584	n2	24	4	100000000	polyglecaprone25	no	MRSA clinical strain
585	n3	0	1	3200000	polyglecaprone25	no	MRSA clinical strain
586	n3	4	2	5400000	polyglecaprone25	no	MRSA clinical strain
587	n3	8	3	12500000	polyglecaprone25	no	MRSA clinical strain
588	n3	24	4	100000000	polyglecaprone25	no	MRSA clinical strain
589	n4	0	1	1700000	polyglecaprone25	no	MRSA clinical strain
590	n4	4	2	5500000	polyglecaprone25	no	MRSA clinical strain
591	n4	8	3	7900000	polyglecaprone25	no	MRSA clinical strain
592	n4	24	4	100000000	polyglecaprone25	no	MRSA clinical strain
593	n5	0	1	670000	polyglecaprone25	no	MRSA clinical strain
594	n5	4	2	1450000	polyglecaprone25	no	MRSA clinical strain
595	n5	8	3	3600000	polyglecaprone25	no	MRSA clinical strain
596	n5	24	4	100000000	polyglecaprone25	no	MRSA clinical strain
597	n6	0	1	390000	polyglecaprone25	no	MRSA clinical strain
598	n6	4	2	8400000	polyglecaprone25	no	MRSA clinical strain
599	n6	8	3	9600000	polyglecaprone25	no	MRSA clinical strain
600	n6	24	4	100000000	polyglecaprone25	no	MRSA clinical strain
601	n1	0	1	290000	polyglactin910	no	MRSA clinical strain
602	n1	4	2	42000000	polyglactin910	no	MRSA clinical strain
603	n1	8	3	88000000	polyglactin910	no	MRSA clinical strain
604	n1	24	4	100000000	polyglactin910	no	MRSA clinical strain
605	n2	0	1	290000	polyglactin910	no	MRSA clinical strain
606	n2	4	2	48000000	polyglactin910	no	MRSA clinical strain
607	n2	8	3	71000000	polyglactin910	no	MRSA clinical strain
608	n2	24	4	100000000	polyglactin910	no	MRSA clinical strain
609	n3	0	1	3200000	polyglactin910	no	MRSA clinical strain
610	n3	4	2	2700000	polyglactin910	no	MRSA clinical strain
611	n3	8	3	3400000	polyglactin910	no	MRSA clinical strain
612	n3	24	4	100000000	polyglactin910	no	MRSA clinical strain
613	n4	0	1	1700000	polyglactin910	no	MRSA clinical strain
614	n4	4	2	2650000	polyglactin910	no	MRSA clinical strain
615	n4	8	3	8450000	polyglactin910	no	MRSA clinical strain
616	n4	24	4	100000000	polyglactin910	no	MRSA clinical strain
617	n5	0	1	670000	polyglactin910	no	MRSA clinical strain

618	n5	4	2	760000	polyglactin910	no	MRSA clinical strain
619	n5	8	3	980000	polyglactin910	no	MRSA clinical strain
620	n5	24	4	100000000	polyglactin910	no	MRSA clinical strain
621	n6	0	1	390000	polyglactin910	no	MRSA clinical strain
622	n6	4	2	640000	polyglactin910	no	MRSA clinical strain
623	n6	8	3	1240000	polyglactin910	no	MRSA clinical strain
624	n6	24	4	100000000	polyglactin910	no	MRSA clinical strain
625	n1	0	1	290000	polydioxanone	no	MRSA clinical strain
626	n1	4	2	45000000	polydioxanone	no	MRSA clinical strain
627	n1	8	3	81000000	polydioxanone	no	MRSA clinical strain
628	n1	24	4	100000000	polydioxanone	no	MRSA clinical strain
629	n2	0	1	290000	polydioxanone	no	MRSA clinical strain
630	n2	4	2	40000000	polydioxanone	no	MRSA clinical strain
631	n2	8	3	68000000	polydioxanone	no	MRSA clinical strain
632	n2	24	4	100000000	polydioxanone	no	MRSA clinical strain
633	n3	0	1	3200000	polydioxanone	no	MRSA clinical strain
634	n3	4	2	5200000	polydioxanone	no	MRSA clinical strain
635	n3	8	3	7600000	polydioxanone	no	MRSA clinical strain
636	n3	24	4	100000000	polydioxanone	no	MRSA clinical strain
637	n4	0	1	1700000	polydioxanone	no	MRSA clinical strain
638	n4	4	2	3600000	polydioxanone	no	MRSA clinical strain
639	n4	8	3	7800000	polydioxanone	no	MRSA clinical strain
640	n4	24	4	100000000	polydioxanone	no	MRSA clinical strain
641	n5	0	1	670000	polydioxanone	no	MRSA clinical strain
642	n5	4	2	970000	polydioxanone	no	MRSA clinical strain
643	n5	8	3	1120000	polydioxanone	no	MRSA clinical strain
644	n5	24	4	100000000	polydioxanone	no	MRSA clinical strain
645	n6	0	1	390000	polydioxanone	no	MRSA clinical strain
646	n6	4	2	541000	polydioxanone	no	MRSA clinical strain
647	n6	8	3	686000	polydioxanone	no	MRSA clinical strain
648	n6	24	4	100000000	polydioxanone	no	MRSA clinical strain
649	n1	0	1	290000	polyglecaprone25	yes	MRSA clinical strain
650	n1	4	2	5300	polyglecaprone25	yes	MRSA clinical strain
651	n1	8	3	1200	polyglecaprone25	yes	MRSA clinical strain
652	n1	24	4	1000	polyglecaprone25	yes	MRSA clinical strain
653	n2	0	1	290000	polyglecaprone25	yes	MRSA clinical strain
654	n2	4	2	5300	polyglecaprone25	yes	MRSA clinical strain
655	n2	8	3	62000	polyglecaprone25	yes	MRSA clinical strain
656	n2	24	4	2100	polyglecaprone25	yes	MRSA clinical strain
657	n3	0	1	3200000	polyglecaprone25	yes	MRSA clinical strain
658	n3	4	2	16000	polyglecaprone25	yes	MRSA clinical strain
659	n3	8	3	3900	polyglecaprone25	yes	MRSA clinical strain
660	n3	24	4	1500	polyglecaprone25	yes	MRSA clinical strain
661	n4	0	1	1700000	polyglecaprone25	yes	MRSA clinical strain
662	n4	4	2	26400	polyglecaprone25	yes	MRSA clinical strain
663	n4	8	3	1640	polyglecaprone25	yes	MRSA clinical strain
664	n4	24	4	990	polyglecaprone25	yes	MRSA clinical strain
665	n5	0	1	670000	polyglecaprone25	yes	MRSA clinical strain
666	n5	4	2	3100	polyglecaprone25	yes	MRSA clinical strain
667	n5	8	3	990	polyglecaprone25	yes	MRSA clinical strain
668	n5	24	4	1120	polyglecaprone25	yes	MRSA clinical strain
669	n6	0	1	390000	polyglecaprone25	yes	MRSA clinical strain
670	n6	4	2	1040	polyglecaprone25	yes	MRSA clinical strain
671	n6	8	3	990	polyglecaprone25	yes	MRSA clinical strain
672	n6	24	4	10600	polyglecaprone25	yes	MRSA clinical strain
673	n1	0	1	290000	polyglactin910	yes	MRSA clinical strain
674	n1	4	2	12000	polyglactin910	yes	MRSA clinical strain
675	n1	8	3	14000	polyglactin910	yes	MRSA clinical strain
676	n1	24	4	54000	polyglactin910	yes	MRSA clinical strain
677	n2	0	1	290000	polyglactin910	yes	MRSA clinical strain
678	n2	4	2	5800	polyglactin910	yes	MRSA clinical strain
679	n2	8	3	83000	polyglactin910	yes	MRSA clinical strain

680	n2	24	4	54000	polyglactin910	yes	MRSA clinical strain
681	n3	0	1	3200000	polyglactin910	yes	MRSA clinical strain
682	n3	4	2	14000	polyglactin910	yes	MRSA clinical strain
683	n3	8	3	9600	polyglactin910	yes	MRSA clinical strain
684	n3	24	4	3000	polyglactin910	yes	MRSA clinical strain
685	n4	0	1	1700000	polyglactin910	yes	MRSA clinical strain
686	n4	4	2	16400	polyglactin910	yes	MRSA clinical strain
687	n4	8	3	8100	polyglactin910	yes	MRSA clinical strain
688	n4	24	4	1650	polyglactin910	yes	MRSA clinical strain
689	n5	0	1	670000	polyglactin910	yes	MRSA clinical strain
690	n5	4	2	2200	polyglactin910	yes	MRSA clinical strain
691	n5	8	3	990	polyglactin910	yes	MRSA clinical strain
692	n5	24	4	1060	polyglactin910	yes	MRSA clinical strain
693	n6	0	1	390000	polyglactin910	yes	MRSA clinical strain
694	n6	4	2	1823	polyglactin910	yes	MRSA clinical strain
695	n6	8	3	990	polyglactin910	yes	MRSA clinical strain
696	n6	24	4	1560	polyglactin910	yes	MRSA clinical strain
697	n1	0	1	290000	polydioxanone	yes	MRSA clinical strain
698	n1	4	2	5800	polydioxanone	yes	MRSA clinical strain
699	n1	8	3	4400	polydioxanone	yes	MRSA clinical strain
700	n1	24	4	32000	polydioxanone	yes	MRSA clinical strain
701	n2	0	1	290000	polydioxanone	yes	MRSA clinical strain
702	n2	4	2	12000	polydioxanone	yes	MRSA clinical strain
703	n2	8	3	12000	polydioxanone	yes	MRSA clinical strain
704	n2	24	4	11000	polydioxanone	yes	MRSA clinical strain
705	n3	0	1	3200000	polydioxanone	yes	MRSA clinical strain
706	n3	4	2	3700	polydioxanone	yes	MRSA clinical strain
707	n3	8	3	990	polydioxanone	yes	MRSA clinical strain
708	n3	24	4	1000	polydioxanone	yes	MRSA clinical strain
709	n4	0	1	1700000	polydioxanone	yes	MRSA clinical strain
710	n4	4	2	16400	polydioxanone	yes	MRSA clinical strain
711	n4	8	3	2900	polydioxanone	yes	MRSA clinical strain
712	n4	24	4	1700	polydioxanone	yes	MRSA clinical strain
713	n5	0	1	670000	polydioxanone	yes	MRSA clinical strain
714	n5	4	2	2200	polydioxanone	yes	MRSA clinical strain
715	n5	8	3	990	polydioxanone	yes	MRSA clinical strain
716	n5	24	4	1070	polydioxanone	yes	MRSA clinical strain
717	n6	0	1	390000	polydioxanone	yes	MRSA clinical strain
718	n6	4	2	1823	polydioxanone	yes	MRSA clinical strain
719	n6	8	3	1100	polydioxanone	yes	MRSA clinical strain
720	n6	24	4	2400	polydioxanone	yes	MRSA clinical strain
721	n1	0	1	500000	polyglecaprone25	no	S. epidermidis ATCC CIP 8155
722	n1	4	2	10000000	polyglecaprone25	no	S. epidermidis ATCC CIP 8155
723	n1	8	3	51000000	polyglecaprone25	no	S. epidermidis ATCC CIP 8155
724	n1	24	4	100000000	polyglecaprone25	no	S. epidermidis ATCC CIP 8155
725	n2	0	1	350000	polyglecaprone25	no	S. epidermidis ATCC CIP 8155
726	n2	4	2	56000000	polyglecaprone25	no	S. epidermidis ATCC CIP 8155
727	n2	8	3	78000000	polyglecaprone25	no	S. epidermidis ATCC CIP 8155
728	n2	24	4	100000000	polyglecaprone25	no	S. epidermidis ATCC CIP 8155
729	n3	0	1	280000	polyglecaprone25	no	S. epidermidis ATCC CIP 8155
730	n3	4	2	5800000	polyglecaprone25	no	S. epidermidis ATCC CIP 8155
731	n3	8	3	100000000	polyglecaprone25	no	S. epidermidis ATCC CIP 8155
732	n3	24	4	100000000	polyglecaprone25	no	S. epidermidis ATCC CIP 8155
733	n4	0	1	640000	polyglecaprone25	no	S. epidermidis ATCC CIP 8155
734	n4	4	2	2700000	polyglecaprone25	no	S. epidermidis ATCC CIP 8155
735	n4	8	3	3800000	polyglecaprone25	no	S. epidermidis ATCC CIP 8155
736	n4	24	4	100000000	polyglecaprone25	no	S. epidermidis ATCC CIP 8155
737	n5	0	1	730000	polyglecaprone25	no	S. epidermidis ATCC CIP 8155
738	n5	4	2	1900000	polyglecaprone25	no	S. epidermidis ATCC CIP 8155
739	n5	8	3	4320000	polyglecaprone25	no	S. epidermidis ATCC CIP 8155
740	n5	24	4	100000000	polyglecaprone25	no	S. epidermidis ATCC CIP 8155
741	n6	0	1	1400000	polyglecaprone25	no	S. epidermidis ATCC CIP 8155

742	n6	4	2	6450000	polyglecaprone25	no	S. epidermidis ATCC CIP 8155
743	n6	8	3	100000000	polyglecaprone25	no	S. epidermidis ATCC CIP 8155
744	n6	24	4	100000000	polyglecaprone25	no	S. epidermidis ATCC CIP 8155
745	n1	0	1	500000	polyglactin910	no	S. epidermidis ATCC CIP 8155
746	n1	4	2	10000000	polyglactin910	no	S. epidermidis ATCC CIP 8155
747	n1	8	3	47000000	polyglactin910	no	S. epidermidis ATCC CIP 8155
748	n1	24	4	100000000	polyglactin910	no	S. epidermidis ATCC CIP 8155
749	n2	0	1	350000	polyglactin910	no	S. epidermidis ATCC CIP 8155
750	n2	4	2	70000000	polyglactin910	no	S. epidermidis ATCC CIP 8155
751	n2	8	3	89000000	polyglactin910	no	S. epidermidis ATCC CIP 8155
752	n2	24	4	100000000	polyglactin910	no	S. epidermidis ATCC CIP 8155
753	n3	0	1	280000	polyglactin910	no	S. epidermidis ATCC CIP 8155
754	n3	4	2	7200000	polyglactin910	no	S. epidermidis ATCC CIP 8155
755	n3	8	3	100000000	polyglactin910	no	S. epidermidis ATCC CIP 8155
756	n3	24	4	100000000	polyglactin910	no	S. epidermidis ATCC CIP 8155
757	n4	0	1	640000	polyglactin910	no	S. epidermidis ATCC CIP 8155
758	n4	4	2	2300000	polyglactin910	no	S. epidermidis ATCC CIP 8155
759	n4	8	3	10600000	polyglactin910	no	S. epidermidis ATCC CIP 8155
760	n4	24	4	100000000	polyglactin910	no	S. epidermidis ATCC CIP 8155
761	n5	0	1	730000	polyglactin910	no	S. epidermidis ATCC CIP 8155
762	n5	4	2	3900000	polyglactin910	no	S. epidermidis ATCC CIP 8155
763	n5	8	3	21800000	polyglactin910	no	S. epidermidis ATCC CIP 8155
764	n5	24	4	100000000	polyglactin910	no	S. epidermidis ATCC CIP 8155
765	n6	0	1	1400000	polyglactin910	no	S. epidermidis ATCC CIP 8155
766	n6	4	2	7200000	polyglactin910	no	S. epidermidis ATCC CIP 8155
767	n6	8	3	100000000	polyglactin910	no	S. epidermidis ATCC CIP 8155
768	n6	24	4	100000000	polyglactin910	no	S. epidermidis ATCC CIP 8155
769	n1	0	1	500000	polydioxanone	no	S. epidermidis ATCC CIP 8155
770	n1	4	2	10000000	polydioxanone	no	S. epidermidis ATCC CIP 8155
771	n1	8	3	39000000	polydioxanone	no	S. epidermidis ATCC CIP 8155
772	n1	24	4	100000000	polydioxanone	no	S. epidermidis ATCC CIP 8155
773	n2	0	1	350000	polydioxanone	no	S. epidermidis ATCC CIP 8155
774	n2	4	2	25000000	polydioxanone	no	S. epidermidis ATCC CIP 8155
775	n2	8	3	60000000	polydioxanone	no	S. epidermidis ATCC CIP 8155
776	n2	24	4	100000000	polydioxanone	no	S. epidermidis ATCC CIP 8155
777	n3	0	1	280000	polydioxanone	no	S. epidermidis ATCC CIP 8155
778	n3	4	2	4500000	polydioxanone	no	S. epidermidis ATCC CIP 8155
779	n3	8	3	100000000	polydioxanone	no	S. epidermidis ATCC CIP 8155
780	n3	24	4	100000000	polydioxanone	no	S. epidermidis ATCC CIP 8155
781	n4	0	1	640000	polydioxanone	no	S. epidermidis ATCC CIP 8155
782	n4	4	2	1960000	polydioxanone	no	S. epidermidis ATCC CIP 8155
783	n4	8	3	3450000	polydioxanone	no	S. epidermidis ATCC CIP 8155
784	n4	24	4	100000000	polydioxanone	no	S. epidermidis ATCC CIP 8155
785	n5	0	1	730000	polydioxanone	no	S. epidermidis ATCC CIP 8155
786	n5	4	2	2100000	polydioxanone	no	S. epidermidis ATCC CIP 8155
787	n5	8	3	9160000	polydioxanone	no	S. epidermidis ATCC CIP 8155
788	n5	24	4	100000000	polydioxanone	no	S. epidermidis ATCC CIP 8155
789	n6	0	1	1400000	polydioxanone	no	S. epidermidis ATCC CIP 8155
790	n6	4	2	3400000	polydioxanone	no	S. epidermidis ATCC CIP 8155
791	n6	8	3	100000000	polydioxanone	no	S. epidermidis ATCC CIP 8155
792	n6	24	4	100000000	polydioxanone	no	S. epidermidis ATCC CIP 8155
793	n1	0	1	500000	polyglecaprone25	yes	S. epidermidis ATCC CIP 8155
794	n1	4	2	990	polyglecaprone25	yes	S. epidermidis ATCC CIP 8155
795	n1	8	3	990	polyglecaprone25	yes	S. epidermidis ATCC CIP 8155
796	n1	24	4	990	polyglecaprone25	yes	S. epidermidis ATCC CIP 8155
797	n2	0	1	350000	polyglecaprone25	yes	S. epidermidis ATCC CIP 8155
798	n2	4	2	990	polyglecaprone25	yes	S. epidermidis ATCC CIP 8155
799	n2	8	3	1000	polyglecaprone25	yes	S. epidermidis ATCC CIP 8155
800	n2	24	4	14000	polyglecaprone25	yes	S. epidermidis ATCC CIP 8155
801	n3	0	1	280000	polyglecaprone25	yes	S. epidermidis ATCC CIP 8155
802	n3	4	2	2870	polyglecaprone25	yes	S. epidermidis ATCC CIP 8155
803	n3	8	3	1027	polyglecaprone25	yes	S. epidermidis ATCC CIP 8155

804	n3	24	4	4200	polyglecaprone25	yes	S. epidermidis ATCC CIP 8155
805	n4	0	1	640000	polyglecaprone25	yes	S. epidermidis ATCC CIP 8155
806	n4	4	2	7600	polyglecaprone25	yes	S. epidermidis ATCC CIP 8155
807	n4	8	3	4120	polyglecaprone25	yes	S. epidermidis ATCC CIP 8155
808	n4	24	4	990	polyglecaprone25	yes	S. epidermidis ATCC CIP 8155
809	n5	0	1	730000	polyglecaprone25	yes	S. epidermidis ATCC CIP 8155
810	n5	4	2	29600	polyglecaprone25	yes	S. epidermidis ATCC CIP 8155
811	n5	8	3	990	polyglecaprone25	yes	S. epidermidis ATCC CIP 8155
812	n5	24	4	1180	polyglecaprone25	yes	S. epidermidis ATCC CIP 8155
813	n6	0	1	1400000	polyglecaprone25	yes	S. epidermidis ATCC CIP 8155
814	n6	4	2	40000	polyglecaprone25	yes	S. epidermidis ATCC CIP 8155
815	n6	8	3	990	polyglecaprone25	yes	S. epidermidis ATCC CIP 8155
816	n6	24	4	1460	polyglecaprone25	yes	S. epidermidis ATCC CIP 8155
817	n1	0	1	350000	polyglactin910	yes	S. epidermidis ATCC CIP 8155
818	n1	4	2	990	polyglactin910	yes	S. epidermidis ATCC CIP 8155
819	n1	8	3	2000	polyglactin910	yes	S. epidermidis ATCC CIP 8155
820	n1	24	4	7700	polyglactin910	yes	S. epidermidis ATCC CIP 8155
821	n2	0	1	350000	polyglactin910	yes	S. epidermidis ATCC CIP 8155
822	n2	4	2	1080	polyglactin910	yes	S. epidermidis ATCC CIP 8155
823	n2	8	3	4100	polyglactin910	yes	S. epidermidis ATCC CIP 8155
824	n2	24	4	6800	polyglactin910	yes	S. epidermidis ATCC CIP 8155
825	n3	0	1	280000	polyglactin910	yes	S. epidermidis ATCC CIP 8155
826	n3	4	2	990	polyglactin910	yes	S. epidermidis ATCC CIP 8155
827	n3	8	3	990	polyglactin910	yes	S. epidermidis ATCC CIP 8155
828	n3	24	4	4000	polyglactin910	yes	S. epidermidis ATCC CIP 8155
829	n4	0	1	640000	polyglactin910	yes	S. epidermidis ATCC CIP 8155
830	n4	4	2	930	polyglactin910	yes	S. epidermidis ATCC CIP 8155
831	n4	8	3	990	polyglactin910	yes	S. epidermidis ATCC CIP 8155
832	n4	24	4	2300	polyglactin910	yes	S. epidermidis ATCC CIP 8155
833	n5	0	1	730000	polyglactin910	yes	S. epidermidis ATCC CIP 8155
834	n5	4	2	270	polyglactin910	yes	S. epidermidis ATCC CIP 8155
835	n5	8	3	290	polyglactin910	yes	S. epidermidis ATCC CIP 8155
836	n5	24	4	3640	polyglactin910	yes	S. epidermidis ATCC CIP 8155
837	n6	0	1	1400000	polyglactin910	yes	S. epidermidis ATCC CIP 8155
838	n6	4	2	21400	polyglactin910	yes	S. epidermidis ATCC CIP 8155
839	n6	8	3	1700	polyglactin910	yes	S. epidermidis ATCC CIP 8155
840	n6	24	4	6200	polyglactin910	yes	S. epidermidis ATCC CIP 8155
841	n1	0	1	500000	polydioxanone	yes	S. epidermidis ATCC CIP 8155
842	n1	4	2	1300	polydioxanone	yes	S. epidermidis ATCC CIP 8155
843	n1	8	3	990	polydioxanone	yes	S. epidermidis ATCC CIP 8155
844	n1	24	4	990	polydioxanone	yes	S. epidermidis ATCC CIP 8155
845	n2	0	1	350000	polydioxanone	yes	S. epidermidis ATCC CIP 8155
846	n2	4	2	990	polydioxanone	yes	S. epidermidis ATCC CIP 8155
847	n2	8	3	990	polydioxanone	yes	S. epidermidis ATCC CIP 8155
848	n2	24	4	6800	polydioxanone	yes	S. epidermidis ATCC CIP 8155
849	n3	0	1	280000	polydioxanone	yes	S. epidermidis ATCC CIP 8155
850	n3	4	2	990	polydioxanone	yes	S. epidermidis ATCC CIP 8155
851	n3	8	3	990	polydioxanone	yes	S. epidermidis ATCC CIP 8155
852	n3	24	4	7500	polydioxanone	yes	S. epidermidis ATCC CIP 8155
853	n4	0	1	640000	polydioxanone	yes	S. epidermidis ATCC CIP 8155
854	n4	4	2	2300	polydioxanone	yes	S. epidermidis ATCC CIP 8155
855	n4	8	3	3400	polydioxanone	yes	S. epidermidis ATCC CIP 8155
856	n4	24	4	990	polydioxanone	yes	S. epidermidis ATCC CIP 8155
857	n5	0	1	730000	polydioxanone	yes	S. epidermidis ATCC CIP 8155
858	n5	4	2	9000	polydioxanone	yes	S. epidermidis ATCC CIP 8155
859	n5	8	3	150	polydioxanone	yes	S. epidermidis ATCC CIP 8155
860	n5	24	4	2000	polydioxanone	yes	S. epidermidis ATCC CIP 8155
861	n6	0	1	1400000	polydioxanone	yes	S. epidermidis ATCC CIP 8155
862	n6	4	2	41800	polydioxanone	yes	S. epidermidis ATCC CIP 8155
863	n6	8	3	9560	polydioxanone	yes	S. epidermidis ATCC CIP 8155
864	n6	24	4	2640	polydioxanone	yes	S. epidermidis ATCC CIP 8155
865	n1	0	1	460000	polyglecaprone25	no	C. albicans ATCC 10231

866	n1	4	2	2000000	polyglecaprone25	no	C. albicans ATCC 10231
867	n1	8	3	40000000	polyglecaprone25	no	C. albicans ATCC 10231
868	n1	24	4	100000000	polyglecaprone25	no	C. albicans ATCC 10231
869	n2	0	1	680000	polyglecaprone25	no	C. albicans ATCC 10231
870	n2	4	2	6900000	polyglecaprone25	no	C. albicans ATCC 10231
871	n2	8	3	8400000	polyglecaprone25	no	C. albicans ATCC 10231
872	n2	24	4	100000000	polyglecaprone25	no	C. albicans ATCC 10231
873	n3	0	1	27000	polyglecaprone25	no	C. albicans ATCC 10231
874	n3	4	2	360000	polyglecaprone25	no	C. albicans ATCC 10231
875	n3	8	3	950000	polyglecaprone25	no	C. albicans ATCC 10231
876	n3	24	4	100000000	polyglecaprone25	no	C. albicans ATCC 10231
877	n4	0	1	420000	polyglecaprone25	no	C. albicans ATCC 10231
878	n4	4	2	158000	polyglecaprone25	no	C. albicans ATCC 10231
879	n4	8	3	632000	polyglecaprone25	no	C. albicans ATCC 10231
880	n4	24	4	100000000	polyglecaprone25	no	C. albicans ATCC 10231
881	n5	0	1	2300000	polyglecaprone25	no	C. albicans ATCC 10231
882	n5	4	2	4020000	polyglecaprone25	no	C. albicans ATCC 10231
883	n5	8	3	2740000	polyglecaprone25	no	C. albicans ATCC 10231
884	n5	24	4	100000000	polyglecaprone25	no	C. albicans ATCC 10231
885	n6	0	1	87000	polyglecaprone25	no	C. albicans ATCC 10231
886	n6	4	2	195000	polyglecaprone25	no	C. albicans ATCC 10231
887	n6	8	3	521000	polyglecaprone25	no	C. albicans ATCC 10231
888	n6	24	4	100000000	polyglecaprone25	no	C. albicans ATCC 10231
889	n1	0	1	460000	polyglactin910	no	C. albicans ATCC 10231
890	n1	4	2	2000000	polyglactin910	no	C. albicans ATCC 10231
891	n1	8	3	40000000	polyglactin910	no	C. albicans ATCC 10231
892	n1	24	4	100000000	polyglactin910	no	C. albicans ATCC 10231
893	n2	0	1	680000	polyglactin910	no	C. albicans ATCC 10231
894	n2	4	2	8000000	polyglactin910	no	C. albicans ATCC 10231
895	n2	8	3	9100000	polyglactin910	no	C. albicans ATCC 10231
896	n2	24	4	100000000	polyglactin910	no	C. albicans ATCC 10231
897	n3	0	1	27000	polyglactin910	no	C. albicans ATCC 10231
898	n3	4	2	132000	polyglactin910	no	C. albicans ATCC 10231
899	n3	8	3	428000	polyglactin910	no	C. albicans ATCC 10231
900	n3	24	4	100000000	polyglactin910	no	C. albicans ATCC 10231
901	n4	0	1	420000	polyglactin910	no	C. albicans ATCC 10231
902	n4	4	2	2700000	polyglactin910	no	C. albicans ATCC 10231
903	n4	8	3	3140000	polyglactin910	no	C. albicans ATCC 10231
904	n4	24	4	100000000	polyglactin910	no	C. albicans ATCC 10231
905	n5	0	1	2300000	polyglactin910	no	C. albicans ATCC 10231
906	n5	4	2	5870000	polyglactin910	no	C. albicans ATCC 10231
907	n5	8	3	12000000	polyglactin910	no	C. albicans ATCC 10231
908	n5	24	4	100000000	polyglactin910	no	C. albicans ATCC 10231
909	n6	0	1	87000	polyglactin910	no	C. albicans ATCC 10231
910	n6	4	2	96000	polyglactin910	no	C. albicans ATCC 10231
911	n6	8	3	154000	polyglactin910	no	C. albicans ATCC 10231
912	n6	24	4	100000000	polyglactin910	no	C. albicans ATCC 10231
913	n1	0	1	460000	polydioxanone	no	C. albicans ATCC 10231
914	n1	4	2	2000000	polydioxanone	no	C. albicans ATCC 10231
915	n1	8	3	40000000	polydioxanone	no	C. albicans ATCC 10231
916	n1	24	4	100000000	polydioxanone	no	C. albicans ATCC 10231
917	n2	0	1	680000	polydioxanone	no	C. albicans ATCC 10231
918	n2	4	2	4300000	polydioxanone	no	C. albicans ATCC 10231
919	n2	8	3	6000000	polydioxanone	no	C. albicans ATCC 10231
920	n2	24	4	100000000	polydioxanone	no	C. albicans ATCC 10231
921	n3	0	1	27000	polydioxanone	no	C. albicans ATCC 10231
922	n3	4	2	136000	polydioxanone	no	C. albicans ATCC 10231
923	n3	8	3	541000	polydioxanone	no	C. albicans ATCC 10231
924	n3	24	4	100000000	polydioxanone	no	C. albicans ATCC 10231
925	n4	0	1	420000	polydioxanone	no	C. albicans ATCC 10231
926	n4	4	2	1470000	polydioxanone	no	C. albicans ATCC 10231
927	n4	8	3	3260000	polydioxanone	no	C. albicans ATCC 10231

928	n4	24	4	100000000	polydioxanone	no	C. albicans ATCC 10231
929	n5	0	1	2300000	polydioxanone	no	C. albicans ATCC 10231
930	n5	4	2	6170000	polydioxanone	no	C. albicans ATCC 10231
931	n5	8	3	10700000	polydioxanone	no	C. albicans ATCC 10231
932	n5	24	4	100000000	polydioxanone	no	C. albicans ATCC 10231
933	n6	0	1	87000	polydioxanone	no	C. albicans ATCC 10231
934	n6	4	2	165000	polydioxanone	no	C. albicans ATCC 10231
935	n6	8	3	224000	polydioxanone	no	C. albicans ATCC 10231
936	n6	24	4	100000000	polydioxanone	no	C. albicans ATCC 10231
937	n1	0	1	460000	polyglecaprone25	yes	C. albicans ATCC 10231
938	n1	4	2	23000	polyglecaprone25	yes	C. albicans ATCC 10231
939	n1	8	3	68000	polyglecaprone25	yes	C. albicans ATCC 10231
940	n1	24	4	140000	polyglecaprone25	yes	C. albicans ATCC 10231
941	n2	0	1	680000	polyglecaprone25	yes	C. albicans ATCC 10231
942	n2	4	2	990	polyglecaprone25	yes	C. albicans ATCC 10231
943	n2	8	3	53000	polyglecaprone25	yes	C. albicans ATCC 10231
944	n2	24	4	400000	polyglecaprone25	yes	C. albicans ATCC 10231
945	n3	0	1	27000	polyglecaprone25	yes	C. albicans ATCC 10231
946	n3	4	2	1600	polyglecaprone25	yes	C. albicans ATCC 10231
947	n3	8	3	10000	polyglecaprone25	yes	C. albicans ATCC 10231
948	n3	24	4	10000	polyglecaprone25	yes	C. albicans ATCC 10231
949	n4	0	1	420000	polyglecaprone25	yes	C. albicans ATCC 10231
950	n4	4	2	990	polyglecaprone25	yes	C. albicans ATCC 10231
951	n4	8	3	996	polyglecaprone25	yes	C. albicans ATCC 10231
952	n4	24	4	2700	polyglecaprone25	yes	C. albicans ATCC 10231
953	n5	0	1	2300000	polyglecaprone25	yes	C. albicans ATCC 10231
954	n5	4	2	340000	polyglecaprone25	yes	C. albicans ATCC 10231
955	n5	8	3	14100	polyglecaprone25	yes	C. albicans ATCC 10231
956	n5	24	4	41700	polyglecaprone25	yes	C. albicans ATCC 10231
957	n6	0	1	87000	polyglecaprone25	yes	C. albicans ATCC 10231
958	n6	4	2	990	polyglecaprone25	yes	C. albicans ATCC 10231
959	n6	8	3	1000	polyglecaprone25	yes	C. albicans ATCC 10231
960	n6	24	4	1040	polyglecaprone25	yes	C. albicans ATCC 10231
961	n1	0	1	460000	polyglactin910	yes	C. albicans ATCC 10231
962	n1	4	2	12000	polyglactin910	yes	C. albicans ATCC 10231
963	n1	8	3	35000	polyglactin910	yes	C. albicans ATCC 10231
964	n1	24	4	90000	polyglactin910	yes	C. albicans ATCC 10231
965	n2	0	1	680000	polyglactin910	yes	C. albicans ATCC 10231
966	n2	4	2	990	polyglactin910	yes	C. albicans ATCC 10231
967	n2	8	3	2200	polyglactin910	yes	C. albicans ATCC 10231
968	n2	24	4	18000	polyglactin910	yes	C. albicans ATCC 10231
969	n3	0	1	27000	polyglactin910	yes	C. albicans ATCC 10231
970	n3	4	2	4800	polyglactin910	yes	C. albicans ATCC 10231
971	n3	8	3	5120	polyglactin910	yes	C. albicans ATCC 10231
972	n3	24	4	100000	polyglactin910	yes	C. albicans ATCC 10231
973	n4	0	1	420000	polyglactin910	yes	C. albicans ATCC 10231
974	n4	4	2	990	polyglactin910	yes	C. albicans ATCC 10231
975	n4	8	3	1450	polyglactin910	yes	C. albicans ATCC 10231
976	n4	24	4	1700	polyglactin910	yes	C. albicans ATCC 10231
977	n5	0	1	2300000	polyglactin910	yes	C. albicans ATCC 10231
978	n5	4	2	11800	polyglactin910	yes	C. albicans ATCC 10231
979	n5	8	3	2070	polyglactin910	yes	C. albicans ATCC 10231
980	n5	24	4	3140	polyglactin910	yes	C. albicans ATCC 10231
981	n6	0	1	87000	polyglactin910	yes	C. albicans ATCC 10231
982	n6	4	2	2060	polyglactin910	yes	C. albicans ATCC 10231
983	n6	8	3	1065	polyglactin910	yes	C. albicans ATCC 10231
984	n6	24	4	2030	polyglactin910	yes	C. albicans ATCC 10231
985	n1	0	1	460000	polydioxanone	yes	C. albicans ATCC 10231
986	n1	4	2	18000	polydioxanone	yes	C. albicans ATCC 10231
987	n1	8	3	69000	polydioxanone	yes	C. albicans ATCC 10231
988	n1	24	4	270000	polydioxanone	yes	C. albicans ATCC 10231
989	n2	0	1	680000	polydioxanone	yes	C. albicans ATCC 10231

990	n2	4	2	990	polydioxanone	yes	C. albicans ATCC 10231
991	n2	8	3	76000	polydioxanone	yes	C. albicans ATCC 10231
992	n2	24	4	68000	polydioxanone	yes	C. albicans ATCC 10231
993	n3	0	1	27000	polydioxanone	yes	C. albicans ATCC 10231
994	n3	4	2	15600	polydioxanone	yes	C. albicans ATCC 10231
995	n3	8	3	23400	polydioxanone	yes	C. albicans ATCC 10231
996	n3	24	4	72000	polydioxanone	yes	C. albicans ATCC 10231
997	n4	0	1	420000	polydioxanone	yes	C. albicans ATCC 10231
998	n4	4	2	990	polydioxanone	yes	C. albicans ATCC 10231
999	n4	8	3	1320	polydioxanone	yes	C. albicans ATCC 10231
1000	n4	24	4	2700	polydioxanone	yes	C. albicans ATCC 10231
1001	n5	0	1	2300000	polydioxanone	yes	C. albicans ATCC 10231
1002	n5	4	2	7400	polydioxanone	yes	C. albicans ATCC 10231
1003	n5	8	3	6040	polydioxanone	yes	C. albicans ATCC 10231
1004	n5	24	4	5960	polydioxanone	yes	C. albicans ATCC 10231
1005	n6	0	1	87000	polydioxanone	yes	C. albicans ATCC 10231
1006	n6	4	2	1020	polydioxanone	yes	C. albicans ATCC 10231
1007	n6	8	3	2540	polydioxanone	yes	C. albicans ATCC 10231
1008	n6	24	4	5480	polydioxanone	yes	C. albicans ATCC 10231
1009	n1	0	1	190000	polyglecaprone25	no	C. albicans clinical strain
1010	n1	4	2	2000000	polyglecaprone25	no	C. albicans clinical strain
1011	n1	8	3	28000000	polyglecaprone25	no	C. albicans clinical strain
1012	n1	24	4	100000000	polyglecaprone25	no	C. albicans clinical strain
1013	n2	0	1	300000	polyglecaprone25	no	C. albicans clinical strain
1014	n2	4	2	6900000	polyglecaprone25	no	C. albicans clinical strain
1015	n2	8	3	8400000	polyglecaprone25	no	C. albicans clinical strain
1016	n2	24	4	100000000	polyglecaprone25	no	C. albicans clinical strain
1017	n3	0	1	328000	polyglecaprone25	no	C. albicans clinical strain
1018	n3	4	2	520000	polyglecaprone25	no	C. albicans clinical strain
1019	n3	8	3	2100000	polyglecaprone25	no	C. albicans clinical strain
1020	n3	24	4	100000000	polyglecaprone25	no	C. albicans clinical strain
1021	n4	0	1	120000	polyglecaprone25	no	C. albicans clinical strain
1022	n4	4	2	340000	polyglecaprone25	no	C. albicans clinical strain
1023	n4	8	3	760000	polyglecaprone25	no	C. albicans clinical strain
1024	n4	24	4	100000000	polyglecaprone25	no	C. albicans clinical strain
1025	n5	0	1	1300000	polyglecaprone25	no	C. albicans clinical strain
1026	n5	4	2	4320000	polyglecaprone25	no	C. albicans clinical strain
1027	n5	8	3	7120000	polyglecaprone25	no	C. albicans clinical strain
1028	n5	24	4	100000000	polyglecaprone25	no	C. albicans clinical strain
1029	n6	0	1	170000	polyglecaprone25	no	C. albicans clinical strain
1030	n6	4	2	354000	polyglecaprone25	no	C. albicans clinical strain
1031	n6	8	3	824000	polyglecaprone25	no	C. albicans clinical strain
1032	n6	24	4	100000000	polyglecaprone25	no	C. albicans clinical strain
1033	n1	0	1	190000	polyglactin910	no	C. albicans clinical strain
1034	n1	4	2	2000000	polyglactin910	no	C. albicans clinical strain
1035	n1	8	3	28000000	polyglactin910	no	C. albicans clinical strain
1036	n1	24	4	100000000	polyglactin910	no	C. albicans clinical strain
1037	n2	0	1	300000	polyglactin910	no	C. albicans clinical strain
1038	n2	4	2	8000000	polyglactin910	no	C. albicans clinical strain
1039	n2	8	3	9100000	polyglactin910	no	C. albicans clinical strain
1040	n2	24	4	100000000	polyglactin910	no	C. albicans clinical strain
1041	n3	0	1	328000	polyglactin910	no	C. albicans clinical strain
1042	n3	4	2	970000	polyglactin910	no	C. albicans clinical strain
1043	n3	8	3	1320000	polyglactin910	no	C. albicans clinical strain
1044	n3	24	4	100000000	polyglactin910	no	C. albicans clinical strain
1045	n4	0	1	120000	polyglactin910	no	C. albicans clinical strain
1046	n4	4	2	470000	polyglactin910	no	C. albicans clinical strain
1047	n4	8	3	820000	polyglactin910	no	C. albicans clinical strain
1048	n4	24	4	100000000	polyglactin910	no	C. albicans clinical strain
1049	n5	0	1	1300000	polyglactin910	no	C. albicans clinical strain
1050	n5	4	2	3400000	polyglactin910	no	C. albicans clinical strain
1051	n5	8	3	7860000	polyglactin910	no	C. albicans clinical strain

1052	n5	24	4	100000000	polyglactin910	no	C. albicans clinical strain
1053	n6	0	1	170000	polyglactin910	no	C. albicans clinical strain
1054	n6	4	2	310000	polyglactin910	no	C. albicans clinical strain
1055	n6	8	3	680000	polyglactin910	no	C. albicans clinical strain
1056	n6	24	4	100000000	polyglactin910	no	C. albicans clinical strain
1057	n1	0	1	190000	polydioxanone	no	C. albicans clinical strain
1058	n1	4	2	2000000	polydioxanone	no	C. albicans clinical strain
1059	n1	8	3	28000000	polydioxanone	no	C. albicans clinical strain
1060	n1	24	4	100000000	polydioxanone	no	C. albicans clinical strain
1061	n2	0	1	300000	polydioxanone	no	C. albicans clinical strain
1062	n2	4	2	4300000	polydioxanone	no	C. albicans clinical strain
1063	n2	8	3	6000000	polydioxanone	no	C. albicans clinical strain
1064	n2	24	4	100000000	polydioxanone	no	C. albicans clinical strain
1065	n3	0	1	328000	polydioxanone	no	C. albicans clinical strain
1066	n3	4	2	498000	polydioxanone	no	C. albicans clinical strain
1067	n3	8	3	854000	polydioxanone	no	C. albicans clinical strain
1068	n3	24	4	100000000	polydioxanone	no	C. albicans clinical strain
1069	n4	0	1	120000	polydioxanone	no	C. albicans clinical strain
1070	n4	4	2	340000	polydioxanone	no	C. albicans clinical strain
1071	n4	8	3	658000	polydioxanone	no	C. albicans clinical strain
1072	n4	24	4	100000000	polydioxanone	no	C. albicans clinical strain
1073	n5	0	1	1300000	polydioxanone	no	C. albicans clinical strain
1074	n5	4	2	5680000	polydioxanone	no	C. albicans clinical strain
1075	n5	8	3	8540000	polydioxanone	no	C. albicans clinical strain
1076	n5	24	4	100000000	polydioxanone	no	C. albicans clinical strain
1077	n6	0	1	170000	polydioxanone	no	C. albicans clinical strain
1078	n6	4	2	370000	polydioxanone	no	C. albicans clinical strain
1079	n6	8	3	687000	polydioxanone	no	C. albicans clinical strain
1080	n6	24	4	100000000	polydioxanone	no	C. albicans clinical strain
1081	n1	0	1	190000	polyglecaprone25	yes	C. albicans clinical strain
1082	n1	4	2	13000	polyglecaprone25	yes	C. albicans clinical strain
1083	n1	8	3	5900	polyglecaprone25	yes	C. albicans clinical strain
1084	n1	24	4	91000	polyglecaprone25	yes	C. albicans clinical strain
1085	n2	0	1	300000	polyglecaprone25	yes	C. albicans clinical strain
1086	n2	4	2	990	polyglecaprone25	yes	C. albicans clinical strain
1087	n2	8	3	6000	polyglecaprone25	yes	C. albicans clinical strain
1088	n2	24	4	7600	polyglecaprone25	yes	C. albicans clinical strain
1089	n3	0	1	328000	polyglecaprone25	yes	C. albicans clinical strain
1090	n3	4	2	990	polyglecaprone25	yes	C. albicans clinical strain
1091	n3	8	3	1700	polyglecaprone25	yes	C. albicans clinical strain
1092	n3	24	4	2500	polyglecaprone25	yes	C. albicans clinical strain
1093	n4	0	1	120000	polyglecaprone25	yes	C. albicans clinical strain
1094	n4	4	2	990	polyglecaprone25	yes	C. albicans clinical strain
1095	n4	8	3	1080	polyglecaprone25	yes	C. albicans clinical strain
1096	n4	24	4	4300	polyglecaprone25	yes	C. albicans clinical strain
1097	n5	0	1	1300000	polyglecaprone25	yes	C. albicans clinical strain
1098	n5	4	2	52000	polyglecaprone25	yes	C. albicans clinical strain
1099	n5	8	3	2300	polyglecaprone25	yes	C. albicans clinical strain
1100	n5	24	4	3000	polyglecaprone25	yes	C. albicans clinical strain
1101	n6	0	1	170000	polyglecaprone25	yes	C. albicans clinical strain
1102	n6	4	2	2030	polyglecaprone25	yes	C. albicans clinical strain
1103	n6	8	3	1960	polyglecaprone25	yes	C. albicans clinical strain
1104	n6	24	4	2000	polyglecaprone25	yes	C. albicans clinical strain
1105	n1	0	1	190000	polyglactin910	yes	C. albicans clinical strain
1106	n1	4	2	3000	polyglactin910	yes	C. albicans clinical strain
1107	n1	8	3	4600	polyglactin910	yes	C. albicans clinical strain
1108	n1	24	4	14000	polyglactin910	yes	C. albicans clinical strain
1109	n2	0	1	300000	polyglactin910	yes	C. albicans clinical strain
1110	n2	4	2	990	polyglactin910	yes	C. albicans clinical strain
1111	n2	8	3	14000	polyglactin910	yes	C. albicans clinical strain
1112	n2	24	4	26000	polyglactin910	yes	C. albicans clinical strain
1113	n3	0	1	328000	polyglactin910	yes	C. albicans clinical strain

1114	n3	4	2	990	polyglactin910	yes	C. albicans clinical strain
1115	n3	8	3	14000	polyglactin910	yes	C. albicans clinical strain
1116	n3	24	4	26000	polyglactin910	yes	C. albicans clinical strain
1117	n4	0	1	120000	polyglactin910	yes	C. albicans clinical strain
1118	n4	4	2	990	polyglactin910	yes	C. albicans clinical strain
1119	n4	8	3	990	polyglactin910	yes	C. albicans clinical strain
1120	n4	24	4	1060	polyglactin910	yes	C. albicans clinical strain
1121	n5	0	1	1300000	polyglactin910	yes	C. albicans clinical strain
1122	n5	4	2	27000	polyglactin910	yes	C. albicans clinical strain
1123	n5	8	3	2600	polyglactin910	yes	C. albicans clinical strain
1124	n5	24	4	1400	polyglactin910	yes	C. albicans clinical strain
1125	n6	0	1	170000	polyglactin910	yes	C. albicans clinical strain
1126	n6	4	2	3200	polyglactin910	yes	C. albicans clinical strain
1127	n6	8	3	2140	polyglactin910	yes	C. albicans clinical strain
1128	n6	24	4	1240	polyglactin910	yes	C. albicans clinical strain
1129	n1	0	1	190000	polydioxanone	yes	C. albicans clinical strain
1130	n1	4	2	7000	polydioxanone	yes	C. albicans clinical strain
1131	n1	8	3	8600	polydioxanone	yes	C. albicans clinical strain
1132	n1	24	4	10000	polydioxanone	yes	C. albicans clinical strain
1133	n2	0	1	300000	polydioxanone	yes	C. albicans clinical strain
1134	n2	4	2	990	polydioxanone	yes	C. albicans clinical strain
1135	n2	8	3	47000	polydioxanone	yes	C. albicans clinical strain
1136	n2	24	4	28000	polydioxanone	yes	C. albicans clinical strain
1137	n3	0	1	328000	polydioxanone	yes	C. albicans clinical strain
1138	n3	4	2	990	polydioxanone	yes	C. albicans clinical strain
1139	n3	8	3	1080	polydioxanone	yes	C. albicans clinical strain
1140	n3	24	4	850	polydioxanone	yes	C. albicans clinical strain
1141	n4	0	1	120000	polydioxanone	yes	C. albicans clinical strain
1142	n4	4	2	990	polydioxanone	yes	C. albicans clinical strain
1143	n4	8	3	990	polydioxanone	yes	C. albicans clinical strain
1144	n4	24	4	1100	polydioxanone	yes	C. albicans clinical strain
1145	n5	0	1	1300000	polydioxanone	yes	C. albicans clinical strain
1146	n5	4	2	80000	polydioxanone	yes	C. albicans clinical strain
1147	n5	8	3	4100	polydioxanone	yes	C. albicans clinical strain
1148	n5	24	4	3800	polydioxanone	yes	C. albicans clinical strain
1149	n6	0	1	170000	polydioxanone	yes	C. albicans clinical strain
1150	n6	4	2	4560	polydioxanone	yes	C. albicans clinical strain
1151	n6	8	3	3040	polydioxanone	yes	C. albicans clinical strain
1152	n6	24	4	1560	polydioxanone	yes	C. albicans clinical strain
1153	n1	0	1	4100000	polyglecaprone25	no	P. aeruginosa clinical strain
1154	n1	4	2	18400000	polyglecaprone25	no	P. aeruginosa clinical strain
1155	n1	8	3	100000000	polyglecaprone25	no	P. aeruginosa clinical strain
1156	n1	24	4	100000000	polyglecaprone25	no	P. aeruginosa clinical strain
1157	n2	0	1	570000	polyglecaprone25	no	P. aeruginosa clinical strain
1158	n2	4	2	21500000	polyglecaprone25	no	P. aeruginosa clinical strain
1159	n2	8	3	100000000	polyglecaprone25	no	P. aeruginosa clinical strain
1160	n2	24	4	100000000	polyglecaprone25	no	P. aeruginosa clinical strain
1161	n3	0	1	160000	polyglecaprone25	no	P. aeruginosa clinical strain
1162	n3	4	2	6400000	polyglecaprone25	no	P. aeruginosa clinical strain
1163	n3	8	3	78000000	polyglecaprone25	no	P. aeruginosa clinical strain
1164	n3	24	4	100000000	polyglecaprone25	no	P. aeruginosa clinical strain
1165	n4	0	1	270000	polyglecaprone25	no	P. aeruginosa clinical strain
1166	n4	4	2	3400000	polyglecaprone25	no	P. aeruginosa clinical strain
1167	n4	8	3	7200000	polyglecaprone25	no	P. aeruginosa clinical strain
1168	n4	24	4	100000000	polyglecaprone25	no	P. aeruginosa clinical strain
1169	n5	0	1	310000	polyglecaprone25	no	P. aeruginosa clinical strain
1170	n5	4	2	2700000	polyglecaprone25	no	P. aeruginosa clinical strain
1171	n5	8	3	6400000	polyglecaprone25	no	P. aeruginosa clinical strain
1172	n5	24	4	100000000	polyglecaprone25	no	P. aeruginosa clinical strain
1173	n6	0	1	86000	polyglecaprone25	no	P. aeruginosa clinical strain
1174	n6	4	2	125000	polyglecaprone25	no	P. aeruginosa clinical strain
1175	n6	8	3	450000	polyglecaprone25	no	P. aeruginosa clinical strain

1176	n6	24	4	100000000	polyglecaprone25	no	P. aeruginosa clinical strain
1177	n1	0	1	4100000	polyglactin910	no	P. aeruginosa clinical strain
1178	n1	4	2	34000000	polyglactin910	no	P. aeruginosa clinical strain
1179	n1	8	3	100000000	polyglactin910	no	P. aeruginosa clinical strain
1180	n1	24	4	100000000	polyglactin910	no	P. aeruginosa clinical strain
1181	n2	0	1	570000	polyglactin910	no	P. aeruginosa clinical strain
1182	n2	4	2	38000000	polyglactin910	no	P. aeruginosa clinical strain
1183	n2	8	3	100000000	polyglactin910	no	P. aeruginosa clinical strain
1184	n2	24	4	100000000	polyglactin910	no	P. aeruginosa clinical strain
1185	n3	0	1	160000	polyglactin910	no	P. aeruginosa clinical strain
1186	n3	4	2	2640000	polyglactin910	no	P. aeruginosa clinical strain
1187	n3	8	3	6500000	polyglactin910	no	P. aeruginosa clinical strain
1188	n3	24	4	100000000	polyglactin910	no	P. aeruginosa clinical strain
1189	n4	0	1	270000	polyglactin910	no	P. aeruginosa clinical strain
1190	n4	4	2	3200000	polyglactin910	no	P. aeruginosa clinical strain
1191	n4	8	3	5100000	polyglactin910	no	P. aeruginosa clinical strain
1192	n4	24	4	100000000	polyglactin910	no	P. aeruginosa clinical strain
1193	n5	0	1	310000	polyglactin910	no	P. aeruginosa clinical strain
1194	n5	4	2	4800000	polyglactin910	no	P. aeruginosa clinical strain
1195	n5	8	3	8700000	polyglactin910	no	P. aeruginosa clinical strain
1196	n5	24	4	100000000	polyglactin910	no	P. aeruginosa clinical strain
1197	n6	0	1	86000	polyglactin910	no	P. aeruginosa clinical strain
1198	n6	4	2	270000	polyglactin910	no	P. aeruginosa clinical strain
1199	n6	8	3	6600000	polyglactin910	no	P. aeruginosa clinical strain
1200	n6	24	4	100000000	polyglactin910	no	P. aeruginosa clinical strain
1201	n1	0	1	4100000	polydioxanone	no	P. aeruginosa clinical strain
1202	n1	4	2	21000000	polydioxanone	no	P. aeruginosa clinical strain
1203	n1	8	3	100000000	polydioxanone	no	P. aeruginosa clinical strain
1204	n1	24	4	100000000	polydioxanone	no	P. aeruginosa clinical strain
1205	n2	0	1	570000	polydioxanone	no	P. aeruginosa clinical strain
1206	n2	4	2	24700000	polydioxanone	no	P. aeruginosa clinical strain
1207	n2	8	3	100000000	polydioxanone	no	P. aeruginosa clinical strain
1208	n2	24	4	100000000	polydioxanone	no	P. aeruginosa clinical strain
1209	n3	0	1	160000	polydioxanone	no	P. aeruginosa clinical strain
1210	n3	4	2	3900000	polydioxanone	no	P. aeruginosa clinical strain
1211	n3	8	3	46200000	polydioxanone	no	P. aeruginosa clinical strain
1212	n3	24	4	100000000	polydioxanone	no	P. aeruginosa clinical strain
1213	n4	0	1	270000	polydioxanone	no	P. aeruginosa clinical strain
1214	n4	4	2	2100000	polydioxanone	no	P. aeruginosa clinical strain
1215	n4	8	3	13000000	polydioxanone	no	P. aeruginosa clinical strain
1216	n4	24	4	100000000	polydioxanone	no	P. aeruginosa clinical strain
1217	n5	0	1	310000	polydioxanone	no	P. aeruginosa clinical strain
1218	n5	4	2	790000	polydioxanone	no	P. aeruginosa clinical strain
1219	n5	8	3	10000000	polydioxanone	no	P. aeruginosa clinical strain
1220	n5	24	4	100000000	polydioxanone	no	P. aeruginosa clinical strain
1221	n6	0	1	86000	polydioxanone	no	P. aeruginosa clinical strain
1222	n6	4	2	1320000	polydioxanone	no	P. aeruginosa clinical strain
1223	n6	8	3	2600000	polydioxanone	no	P. aeruginosa clinical strain
1224	n6	24	4	100000000	polydioxanone	no	P. aeruginosa clinical strain
1225	n1	0	1	4100000	polyglecaprone25	yes	P. aeruginosa clinical strain
1226	n1	4	2	11400000	polyglecaprone25	yes	P. aeruginosa clinical strain
1227	n1	8	3	100000000	polyglecaprone25	yes	P. aeruginosa clinical strain
1228	n1	24	4	100000000	polyglecaprone25	yes	P. aeruginosa clinical strain
1229	n2	0	1	570000	polyglecaprone25	yes	P. aeruginosa clinical strain
1230	n2	4	2	860000	polyglecaprone25	yes	P. aeruginosa clinical strain
1231	n2	8	3	100000000	polyglecaprone25	yes	P. aeruginosa clinical strain
1232	n2	24	4	100000000	polyglecaprone25	yes	P. aeruginosa clinical strain
1233	n3	0	1	160000	polyglecaprone25	yes	P. aeruginosa clinical strain
1234	n3	4	2	498000	polyglecaprone25	yes	P. aeruginosa clinical strain
1235	n3	8	3	70000000	polyglecaprone25	yes	P. aeruginosa clinical strain
1236	n3	24	4	100000000	polyglecaprone25	yes	P. aeruginosa clinical strain
1237	n4	0	1	270000	polyglecaprone25	yes	P. aeruginosa clinical strain

1238	n4	4	2	4800000	polyglecaprone25	yes	P. aeruginosa clinical strain
1239	n4	8	3	21000000	polyglecaprone25	yes	P. aeruginosa clinical strain
1240	n4	24	4	100000000	polyglecaprone25	yes	P. aeruginosa clinical strain
1241	n5	0	1	310000	polyglecaprone25	yes	P. aeruginosa clinical strain
1242	n5	4	2	1700000	polyglecaprone25	yes	P. aeruginosa clinical strain
1243	n5	8	3	26000000	polyglecaprone25	yes	P. aeruginosa clinical strain
1244	n5	24	4	100000000	polyglecaprone25	yes	P. aeruginosa clinical strain
1245	n6	0	1	86000	polyglecaprone25	yes	P. aeruginosa clinical strain
1246	n6	4	2	240000	polyglecaprone25	yes	P. aeruginosa clinical strain
1247	n6	8	3	1400000	polyglecaprone25	yes	P. aeruginosa clinical strain
1248	n6	24	4	100000000	polyglecaprone25	yes	P. aeruginosa clinical strain
1249	n1	0	1	4100000	polyglactin910	yes	P. aeruginosa clinical strain
1250	n1	4	2	35200000	polyglactin910	yes	P. aeruginosa clinical strain
1251	n1	8	3	100000000	polyglactin910	yes	P. aeruginosa clinical strain
1252	n1	24	4	100000000	polyglactin910	yes	P. aeruginosa clinical strain
1253	n2	0	1	570000	polyglactin910	yes	P. aeruginosa clinical strain
1254	n2	4	2	630000	polyglactin910	yes	P. aeruginosa clinical strain
1255	n2	8	3	97000000	polyglactin910	yes	P. aeruginosa clinical strain
1256	n2	24	4	100000000	polyglactin910	yes	P. aeruginosa clinical strain
1257	n3	0	1	160000	polyglactin910	yes	P. aeruginosa clinical strain
1258	n3	4	2	340000	polyglactin910	yes	P. aeruginosa clinical strain
1259	n3	8	3	18000000	polyglactin910	yes	P. aeruginosa clinical strain
1260	n3	24	4	100000000	polyglactin910	yes	P. aeruginosa clinical strain
1261	n4	0	1	270000	polyglactin910	yes	P. aeruginosa clinical strain
1262	n4	4	2	3700000	polyglactin910	yes	P. aeruginosa clinical strain
1263	n4	8	3	41000000	polyglactin910	yes	P. aeruginosa clinical strain
1264	n4	24	4	100000000	polyglactin910	yes	P. aeruginosa clinical strain
1265	n5	0	1	310000	polyglactin910	yes	P. aeruginosa clinical strain
1266	n5	4	2	280000	polyglactin910	yes	P. aeruginosa clinical strain
1267	n5	8	3	1400000	polyglactin910	yes	P. aeruginosa clinical strain
1268	n5	24	4	100000000	polyglactin910	yes	P. aeruginosa clinical strain
1269	n6	0	1	86000	polyglactin910	yes	P. aeruginosa clinical strain
1270	n6	4	2	540000	polyglactin910	yes	P. aeruginosa clinical strain
1271	n6	8	3	2100000	polyglactin910	yes	P. aeruginosa clinical strain
1272	n6	24	4	100000000	polyglactin910	yes	P. aeruginosa clinical strain
1273	n1	0	1	4100000	polydioxanone	yes	P. aeruginosa clinical strain
1274	n1	4	2	26000000	polydioxanone	yes	P. aeruginosa clinical strain
1275	n1	8	3	100000000	polydioxanone	yes	P. aeruginosa clinical strain
1276	n1	24	4	100000000	polydioxanone	yes	P. aeruginosa clinical strain
1277	n2	0	1	570000	polydioxanone	yes	P. aeruginosa clinical strain
1278	n2	4	2	740000	polydioxanone	yes	P. aeruginosa clinical strain
1279	n2	8	3	5610000	polydioxanone	yes	P. aeruginosa clinical strain
1280	n2	24	4	100000000	polydioxanone	yes	P. aeruginosa clinical strain
1281	n3	0	1	160000	polydioxanone	yes	P. aeruginosa clinical strain
1282	n3	4	2	485000	polydioxanone	yes	P. aeruginosa clinical strain
1283	n3	8	3	6740000	polydioxanone	yes	P. aeruginosa clinical strain
1284	n3	24	4	100000000	polydioxanone	yes	P. aeruginosa clinical strain
1285	n4	0	1	270000	polydioxanone	yes	P. aeruginosa clinical strain
1286	n4	4	2	7500000	polydioxanone	yes	P. aeruginosa clinical strain
1287	n4	8	3	14000000	polydioxanone	yes	P. aeruginosa clinical strain
1288	n4	24	4	100000000	polydioxanone	yes	P. aeruginosa clinical strain
1289	n5	0	1	86000	polydioxanone	yes	P. aeruginosa clinical strain
1290	n5	4	2	145000	polydioxanone	yes	P. aeruginosa clinical strain
1291	n5	8	3	4960000	polydioxanone	yes	P. aeruginosa clinical strain
1292	n5	24	4	100000000	polydioxanone	yes	P. aeruginosa clinical strain
1293	n6	0	1	86000	polydioxanone	yes	P. aeruginosa clinical strain
1294	n6	4	2	145000	polydioxanone	yes	P. aeruginosa clinical strain
1295	n6	8	3	4960000	polydioxanone	yes	P. aeruginosa clinical strain
1296	n6	24	4	100000000	polydioxanone	yes	P. aeruginosa clinical strain
